# The Molecular Basis of Ubiquitin-Conjugating Enzymes (E2s) as a Potential Target for Cancer Therapy

**DOI:** 10.3390/ijms22073440

**Published:** 2021-03-26

**Authors:** Xiaodi Du, Hongyu Song, Nengxing Shen, Ruiqi Hua, Guangyou Yang

**Affiliations:** Department of Parasitology, College of Veterinary Medicine, Sichuan Agricultural University, Chengdu 611130, China; 2019203033@stu.sicau.edu.cn (X.D.); songhongyu95@outlook.com (H.S.); shennengxing@stu.sicau.edu.cn (N.S.); ruiqihua@stu.sicau.edu.cn (R.H.)

**Keywords:** ubiquitin-conjugating enzymes, E2s, cancer, target, NF-κB, inhibitors

## Abstract

Ubiquitin-conjugating enzymes (E2s) are one of the three enzymes required by the ubiquitin-proteasome pathway to connect activated ubiquitin to target proteins via ubiquitin ligases. E2s determine the connection type of the ubiquitin chains, and different types of ubiquitin chains regulate the stability and activity of substrate proteins. Thus, E2s participate in the regulation of a variety of biological processes. In recent years, the importance of E2s in human health and diseases has been particularly emphasized. Studies have shown that E2s are dysregulated in variety of cancers, thus it might be a potential therapeutic target. However, the molecular basis of E2s as a therapeutic target has not been described systematically. We reviewed this issue from the perspective of the special position and role of E2s in the ubiquitin-proteasome pathway, the structure of E2s and biological processes they are involved in. In addition, the inhibitors and microRNAs targeting E2s are also summarized. This article not only provides a direction for the development of effective drugs but also lays a foundation for further study on this enzyme in the future.

## 1. Introduction

The 2004 Nobel Prize in Chemistry was awarded to three scientists (Aron Ciechanover, Avaram Heshko and Irwin Rose) for their discovery of the mechanism of protein degradation regulated by ubiquitin (Ub). They found the same class of peptides in mammals, bacteria and plants, whose structures and properties were very similar. They named it Ub because of its widespread existence [1]. Ub has the property of forming stable chemical bonds with other proteins to regulate their stability, activity or position [2]. Ub modifies substrate proteins through the ubiquitin-proteasome pathway (UPP), which requires three key enzymes—Ubiquitin-activating enzyme (E1), ubiquitin-conjugating enzyme (E2), and ubiquitin ligase (E3), to hydrolyze the target proteins of ubiquitination in cells to maintain protein homeostasis [3].

Residing at the center of the cascade, E2s largely determine Ub chain topology and are responsible for recruiting E3 ligases and their substrates [4]. The relationship between E2s and human cancer has been studied extensively. Ullah et al. [5] showed that increased expression or mutation of *UBE2O* is common in breast cancer (BC), gastric cancer (GC), renal carcinoma (RC) and ovarian cancer (OC). The UBE2C mRNA or protein can hardly be detected in normal tissues; however, it is abnormally elevated in diseases such as cerebral cancer, lung cancer (LC), leukemia, lymphoma, GC, BC, colon cancer (CC) and hepatocellular carcinoma (HCC) [6,7,8,9]. Even *UBE2C* transgenic mice are more likely to develop spontaneous tumors and tumors induced by carcinogens [10]. Wu et al. [11] found that UBE2N is related to the low overall survival rate of human BC and is directly involved in the process of metastasis, which is necessary for lung colonization and the survival and proliferation of established metastatic lesions. Cases of the abnormal expression of other E2 members in different cancer cells or tissues are also widespread. This not only affects the protein turnover of cancer cells, but also indirectly regulates the proliferation, metastasis, anti-apoptosis and drug resistance of cancer cells by participating in certain biological processes. Although researchers are committed to studying the roles of E2s in various diseases and constantly emphasize the potential of E2s as a therapeutic target, the molecular basis of E2s as a potential therapeutic target for cancer has not been described systematically. A newly published review classified systematically all the reported small molecular inhibitors of the basic components of UPP, including E1, E2, E3, 20s proteasome catalytic core particles and 19s proteasome catalytic core particles, as well as their mechanisms of action and limitations [12]; however, the inhibitors and microRNAs (miRNAs) targeting E2s were not fully summarized. Therefore, we reviewed the literature in the past five years to cover these two aspects. We discuss the molecular basis of E2s as a target for cancer therapy from the special position and functions of E2s in UPP, the structure of E2s and biological processes they are participated in, and the efforts made to develop corresponding inhibitors. Researchers can select E2 as a specific target and consider the possible positive and negative effects according to the biological processes it participates in, allowing them to develop effective inhibitors with few side effects to control the occurrence and development of cancer.

## 2. The Position of E2 in UPP

**Figure 1:**
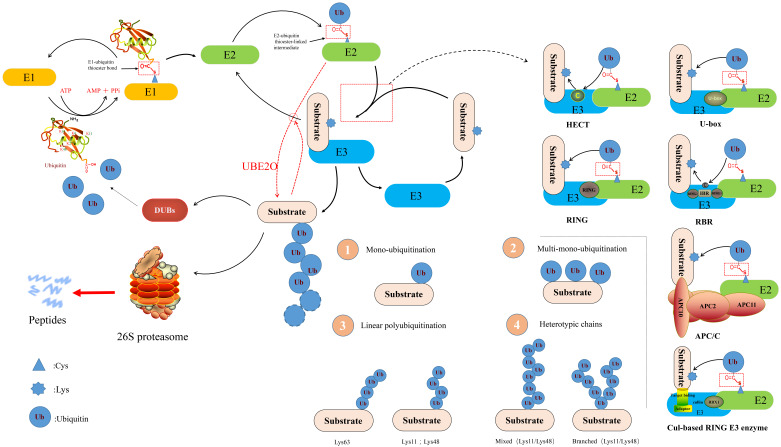
A schematic diagram of the ubiquitin-proteasome pathway (refer to [13]). Ubiquitin (Ub) is activated by ubiquitin-activating enzyme in an adenosine-triphosphate-dependent manner. Ubiquitin-activating enzyme hydrolyzes adenosine-triphosphate and forms a complex with Ub. Subsequently, Ub is transferred to one of many different E2s. In some reactions, E2 (UBE2O) can directly ubiquitinate the substrate, while others need the help of ubiquitin ligases (E3s). E3s are currently categorized into three families, (i) Homologous to E6-associated Protein C-terminus (HECT), (ii) Really Interesting New Gene (RING), and (iii) U-box Domain [25]. The Cullin-RING ligases (CRLs), APC/C ligase, and RING-Between-RING ligases (RBRs) all belong to RING E3s. Ubiquitination of substrates occur as mentioned in the three ways: Monoubiquitination, multi-monoubiquitination, and polyubiquitination (including linear polyubiquitination and branched polyubiquitination).

The UPP comprises Ub, E1, E2, E3, 26S proteasome, deubiquitinating enzymes (DUBs) and target proteins [13]. It exists widely in eukaryotes as well as in prokaryotes, and is an important pathway for selective protein degradation in organisms. The UPP involves two consecutive processes: The ubiquitination system ubiquitinates the substrate proteins and 26S proteasome degrades ubiquitinated target proteins [14]. First, under the energy supply of adenosine-triphosphate (ATP), the C-glycine residue of Ub forms a high energy thioester bond with the cysteine residue of E1. Later, E1 connects the activated Ub to E2 through the intermediate product of Ub adenylation to form the E2-Ub sulfhydryl ester. Although Ub can be transferred directly or indirectly from E2 to the substrate, E3 is usually needed to identify specific substrates. Through a E3 enzyme, Ub is transferred from the thioester intermediate to the ε amino group of the target protein (lysine residue) to form an isopeptide bond and then subsequently activates the Ub to connect the 48th lysine residue of the previous Ub molecule to form a signal. This polyubiquitin (polyUb) chain is recognized and degraded by proteasome [15,16] (Figure 1).

E2 are multi-functional enzymes that primarily engage in two types of reactions with a simple active site in the UPP: (1) Transthiolation (transfer from a thioester to a thiol group) and (2) Aminolysis (transfer from a thioester to an amino group). Most E2s can interact with E1 and one or more E3s [17], providing a binding platform for E1, E3, and activated Ub/Ub-like (Ubl). Some E2s also play a role outside the traditional Ub transport pathway by regulating the activity of other enzymes [18]. It should be pointed out that Ub contains seven different lysine residues (Lys6, Lys11, Lys27, Lys29, Lys33, Lys48, and Lys63), any of which can be covalently linked with other Ub molecules. Besides, the N-terminal (N-) methionine (M1) of Ub is also a site for covalent binding of other Ubs to form M1-linked linear ubiquitylation [19]. So, the type of these Ub chains and the fate of substrate proteins are determined by E2s. PolyUbs can be formed through the same Ub linkage (homotypic chains) or a combination of different lysine linkages, resulting in mixed or branched structures (heterotypic chains). It is only through recognition of these different Ub structures by Ub-binding proteins (UBPs) that the intracellular fate of the protein is determined [20]. A branched Lys11/Lys48 chain in heterotypic chains is considered to be a better signal for proteasome degradation [21]. Generally speaking, Lys48 and Lys11 chains are further involved in proteasome degradation. Proteasome shuttling factors preferentially bind Lys48 chains compared with Lys11 chains. M1-linked ubiquitin chains, Lys63 and Lys6 chains are usually involved in non-proteolytic processes, such as immune homoeostasis [22], DNA repair, signal transduction, and endosomal-lysosomal degradation [23]. The Lys11 chain is less studied than the Lys48 or Lys63 chain, but it seems to serve as a degradation signal for anaphase-promoting complex/cyclosome (APC/C) substrates in the regulation of cell division [24]. The functions of other connection types of Ub chains are not entirely clear.

## 3. The Structure and Classification of E2

**Figure 2:**
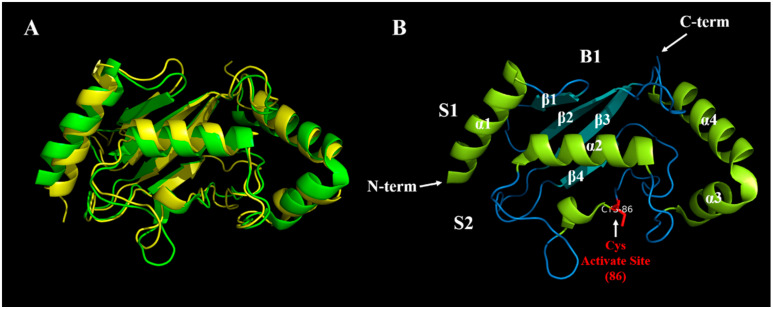
(**A**) The alignment of UBE2T and UBE2I (Ubl). The three dimensional (3D) structure of UBE2T is shown in green, while UBE2I is shown in yellow, indicating that the structure of E2 family is conserved. UBE2T has a C-terminal extension, which belongs to Class III, while UBE2I has no C- and N- extensions, and so belongs to Class I, which are consistent with the summary shown in Table 1 (**B**) The 3D structure of UBE2T. We marked four α-helixes and four β-folds with white letters. N-term and C-term refer to the N- and C-termini of UBE2T, respectively. The main sites of interaction between UBE2T and E1 and E3 are S1 and S2, respectively, and the active site cysteine (86) is highlighted in red. B1 is opposite to the active site of UBE2T, which is called the ‘backside.’ Backside binding between E2 and Ub or SUMO contributes to chain building activities or regulates E2 activities via allostery [32]. The 3D structures of UBE2T and UBE2I were downloaded from http://www.rcsb.org/ (accessed on 20 November 2020), numbered 5NGZ and 1A3S, respectively. PyMOL software was used to align them, and the prediction of the active site was carried out using http://www.ebi.ac.uk/interpro/result/InterProScan/iprscan5-R20200618-032207-0413-15289371-p2m/ (accessed on 20 November 2020).

To date, 1635 E2s have been identified in eukaryotic genomes and about 40 E2s in human have been identified to be involved in the transfer of Ub/Ubl proteins (e.g., small ubiquitin-like modifier (SUMO) and NEDD8), while other eukaryotic genome E2 families have 16–35 members [26]. Their names consist of the designation ubiquitin enzyme E2 (UBE2) followed by a serial capital letter according to their consecutive discovery. Most E2s have a highly conserved ubiquitin-conjugating region comprising 150–200 amino acids, known as the UBC domain, which binds Ub/Ubl proteins activated by ATP (Figure 2A). This domain adopts an α/β-fold, typically with four α-helices and a four stranded β-sheets (Figure 2B). The UBC domain is embellished with functionally important insertions, such as UBE2R1 or UBE2G2. Moreover, a few E2s have an additional structured domain linked to their UBC domain (e.g., UBE2K) or are part of large multi-domain proteins (e.g., UBE2O) [27]. Ubiquitin-conjugating enzyme variant (UEV) exhibits high similarity with E2s both in structure and amino acid sequence, but lacks the UBC domain and the active-site cysteine residue, and thus is catalytically inactive [28]. The E2 family has been divided into four classes: Class I, UBC domain only; class II, UBC domain plus an N- extension; class III, UBC plus a C- extension; and class IV, UBC plus both N- and C- extensions [29]. These extra domains not only create E2s of diverse molecular size, including the largest E2, BIRC6 (4857 amino acids), but also can govern intracellular localization, confer regulatory properties, and enable specific interactions with particular E3s [30]. Although the shallow and exposed nature of the catalytic site make it difficult to target the site directly with small molecules, the distinct regions in which E2s are involved in many protein-protein interactions raise the possibility of additional modes of inhibition [31].

## 4. Biological Processes Involving E2s

### 4.1. DNA Repair Pathway

**Figure 3:**
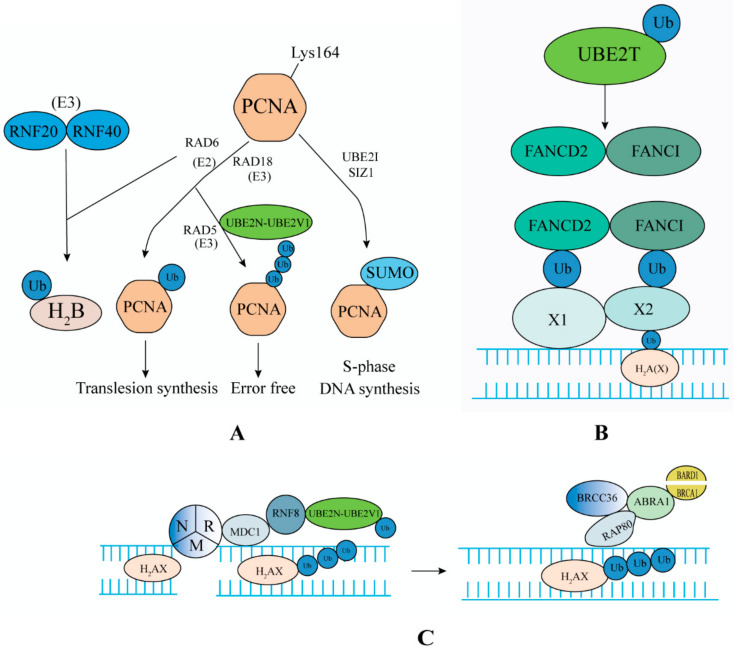
E2s participate in the DNA repair pathway. (**A**) E2 ubiquitinates proliferating cell nuclear antigen (PCNA) to participate in the DNA replication and repair process. (**B**) E2 ubiquitinates the FANCD2 and FANCI complex to participate in the FA pathway. (**C**) E2 ubiquitinates H2A(X) to participate in the DNA replication and repair process. UBE2D3 can also participate in the above (**A**,**C**) processes [151].

Certain proteins are ubiquitinated by radiation or treatment with DNA damaging agents, including proliferating cell nuclear antigen (PCNA), histone H2A and its variant H2A(X), 9-1-1 complex, Fanconi (FA) pathway proteins FANCD2 and FANCI, and replication factor 2 [127] (Figure 3). The pathways of E2s involved in DNA damage repair include: DNA translesion synthesis (TLS) and error free pathway targeting PCNA, DNA double strand break repair (DSBR) and the FA pathway centered on H2A(X) [33]. In addition, some E2 members are involved in other types of DNA repair processes. The SUMOylation of Xerderma pigmentosum C by UBE2I is to participate in nucleotide excision repair (NER) [128]. UBE2I also plays an important role in DNA-protein crosslinks (DPCs) repair by subsequent proteasome hydrolysis of SUMOylation TOP1/TOP2-DPC (Topoisomerase I/II-DNA-protein crosslink), which repairs the inhibition of DNA metabolism caused by the persistence of TOP (Topoisomerase) [129]. RAD6 mutants are sensitive to alkylating agents, and alkylating agent-induced DNA damage is repaired by base excision repair (BER) pathway, indicating that RAD6 may be involved in BER. UBE2K is involved in these two different repair processes [130]. Damaged DNA can lead to cell cycle arrest and induce the process of DNA repair. Once the damage is repaired, the cells that stop the checkpoint will resume the cell cycle. The accumulation of unrepaired DNA usually triggers the activation of multiple cell death or carcinogenic pathways, resulting in cell death, senescence, or cancer [131].

UBE2N cooperates with UBE2V2 to modify PCNA to participate in DNA replication and repair pathways [132]. DNA damage induces monoubiquitination at Lys164 of PCNA, which is catalyzed by UBA1, RAD6, and RAD18 (which also involved in the activation of FA pathway). DNA damage induces nuclear entry of UBE2N and UBE2V2, which are recruited into chromatin by RAD5, where they catalyze PCNA to form a Lys63-linked polyUb chain. RAD6 binds to RAD18 to regulate the TLS pathway of mutant DNA in response to genomic damage, including that induced by chemotherapy and radiotherapy. In the absence of DNA damaging agent, PCNA is modified by SUMO in the S phase, preventing post-replication repair enzymes from being recruited into an inappropriate period of the cell cycle [133] (Figure 3A). UBE2N can also interact with RNF8 and RNF168 to participate in the DNA injury response. OTU domain-containing ubiquitin aldehyde binding protein 1 (OTUB1) regulates DNA repair by hydrolyzing the UBE2N-mediated Lys63 polyUb chain [134].

DSBs can be repaired by homologous recombination (HR) or non-homologous end joining (NHEJ). HR plays an important role in the S/G2 phase of the cell cycle, while NHEJ can function in all phases of the cell cycle [135]. UBE2S was found to be associated with the components of the NHEJ complex and participates in the NHEJ-mediated DNA repair process [136]. The MRN complex (MRE11, RAD50, NBS1) detects DNA DSBs and forms a complex with phosphorylated mediator of DNA damage checkpoint protein 1 (MDC1) and histone H2AX. MDC1 recruits RNF8/UBE2N/UBE2V2, to make H2AX form a Lys63-linked polyUb chain. RAP80 recognizes the Lys63 polyUb chain on H2AX and forms a complex with ABRA1 (Abraxas), BRCC36, breast cancer-1 (BRCA1), and BARD to participate in HR (Figure 3C). OTUB1 inhibits the process via deubiquitination of UBE2N. By collaborating with the heterodimeric E3 ligase RNF20/RNF40, RAD6 catalyzes the monoubiquitination of H2B to participate in the regulation of DNA damage repair [137].

UBE2T (also known as FANCT) is first identified in a case of FA, and catalyzes the monoubiquitination of the FANCD2 and FANCI complex with FANCL (RING-type E3). Then, they cluster in DNA interstrand cross-link (ICL) lesions by binding with unknown X1 or X2 in the DNA repair process [138] (Figure 3B). The RAD18/RAD6 complex is also crucial to activate the FA pathway. When the repair process is completed, the FANCD2 and FANCI complex is deubiquitylated and dissociated from the repaired ICL site by the USP1-UAF1 complex and is then released from the DNA [139]. The destruction of UBE2T expression leads to FA (a DNA repair defect) by affecting the damage repair response of DNA ICL. On the one hand, it increases the risk of early cancer in adults [140]; on the other hand, it increases the sensitivity of cancer cells to cross-linking agents [141]. Lyakhovich [142] showed that knocking down *FANCD2* increased the sensitivity of cancer cells (BC, Bladder, or LC cell lines) to mitomycin C (MMC) and to a lesser extent, to gamma-rays. Importantly, those cell lines with significant *FANCD2* depletion revealed a decreased recurrence capacity. Ramaeker [143] showed that hypoxia can rapidly and forcefully reduce the level of *UBE2T* mRNA in cancer cell lines, thus greatly increasing the sensitivity of cancer cells to MMC therapy. Therefore, to reduce the risk of cancer, it is necessary to ensure stable levels of UBE2T and other proteins involved in the FA pathway. To treat patients with cancer, the combination of DNA cross-linking agents (e.g., MMC, Cisplatin (DDP)) with inhibitors of the FA pathway might kill cancer cells by preventing them from replicating and repairing. UBE2T can also promote the removal of DSBs in the process of DNA HR repair [144].

Overall, in normal cells, E2s are involved in DNA repair and restart of damaged DNA replication forks to maintain genomic integrity and reduce the incidence of cancer [145]. The increased expression and activation of DNA damage response signals and repair genes are the reasons for the resistance of cancer cells to radiotherapy [146]. Downregulation of UBE2S expression in Glioblastoma (GBM) suppressed NHEJ-mediated DSBR, and made GBM cells more sensitive to radiotherapy [136]. RAD6 regulates mutagenic TLS and FA in response to various genomic insults, including chemo and radiation therapy [147]. TZ9 (a RAD6-specific small molecule inhibitor) treatment of triple negative breast cancer (TNBC) and OC cells weakens the DNA repair signal of these cells, thus increasing the sensitivity of drug-resistant cancer cells to carplatin [148]. UBE2B regulates the sensitivity of nasopharyngeal carcinoma (NPC) cells to carmustine (BCNU) by ubiquitination of MGMT (a DNA repair enzyme) [149]. UBE2D3 had the opposite effect, and its downregulation promoted DNA damage repair in esophageal cancer cells and enhanced the radiation resistance of cancer cells [150]. We know that NHEJ-mediated DSBR exists in all stages of the cell cycle, and the repair process is fast but inaccurate. Although HR-mediated DSBR only exists in the S/G2 phase of the cell cycle, the repair process is complex but accurate. Therefore, UBE2S and UBE2N, which are involved in these two processes, can be considered as anticancer targets. In view of the ineffectiveness of radiotherapy and emergence of chemotherapy resistance, it is urgent to develop inhibitors of DNA damage repair signals. Combining the inhibitors with radiotherapy and chemotherapeutic drugs mighty increase the effectiveness of cancer treatment. This requires further study.

### 4.2. Cell Cycle

**Figure 4:**
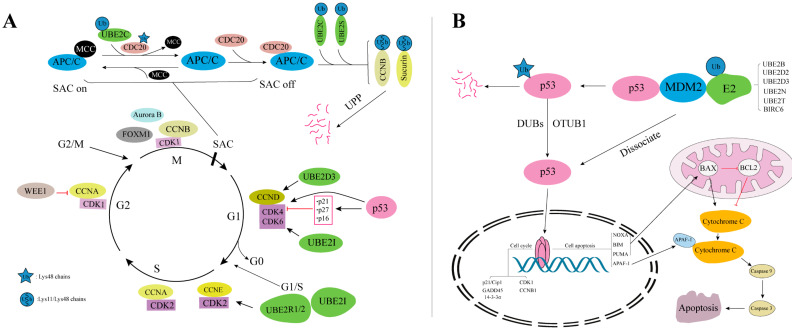
(**A**) E2s participate in the cell cycle process. (**B**) E2s regulate the cell cycle and apoptosis process by ubiquitinating p53. p53 upregulated modulator of apoptosis (PUMA) and BCL2-associated X (BAX) can induce apoptosis through increasing mitochondrial outer membrane permeability.

Cell cycle progression is monitored by a set of checkpoints G1/S, G2/M, and spindle-assembly checkpoint (SAC) that push cells forward from one stage to the next [35], and their transformation depend largely on the UPP (Figure 4A). Kinase inhibition protein p27 (p27^Kip1^), as an inhibitor of cyclin-dependent kinase (CDK), can be ubiquitinated by UBE2D and UBE2R1 in the transition of G1max to S phase, thus alleviating the inhibition of the CDK4/CDK6 complex [152]. The genes regulated by p27^Kip1^ are closely associated with poor cancer survival, indicating that p27^Kip1^ is associated with cancer progression [153]. UBE2D3 is also the initial ubiquitination enzyme for key regulatory molecules such as cell cyclin D1 (CCND1), inhibitor of nuclear factor kappa B (IκB), p53, and MDM2 [154]. UBE2R1 and UBE2R2 participate in the extension of cell cyclin E (CCNE) ubiquitin chain [155]. UBE2C has been proposed to be rate-limiting in the late G1 phase, when it is required to degrade cell cyclin A by APC/C to prevent premature DNA replication [156]. RAD6 influences the transcription of CDK1 by increasing monoubiquitinylation of H2B and trimethylation of H3K4 in the *CDK1* promoter region. Therefore, RAD6 promotes G1/S phase transition and cell proliferation [157]. In addition, SUMO also plays a significant role in the cell cycle, and CDK1, CDK9, CDK11, and CCNE have been identified as targets of SUMO [158]. In GBM cells, SUMOylation of CDK6 can prevent its ubiquitination and degradation, and ensure its existence in the process of G1/S transformation [159]. In the transition of G/M phase, this process is promoted by ubiquitination of WEE1 [160].

SAC ensures an equal distribution of chromosomes to daughter cells during mitosis [161]. Improper SAC results in malignancies or birth defects. APC/C (SAC inhibits APC/C) is also one of the determinants of precise division of genetic material during mitosis [162]. The inhibition of APC/C by SAC is inhibited by the tetramer binding of mitotic checkpoint complex (MCC) formed by cell division cycle 20 (CDC20), MAD2, BUBR1, and BUB3 in the G2/S phase (Figure 4A). UBE2C has been reported to be required to dissociate the MAD2-CDC20 complex by ubiquitylating CDC20, thus releasing the MCC [163]. Inactive APC/C is activated by binding with free CDC20, and then the activated APC/C ubiquitinates securin and cell cyclin B1 (CCNB1) via UBE2C and UBE2S [164]. Subsequently, securin and CCNB1 are ubiquitinated and degraded rapidly, resulting in the transition from metaphase to anaphase of mitosis [165]. The recognition of different ubiquitin chains by proteasomes gives different “priority” to the degradation of substrates. The heteromorphic Lys11/Lys48 chains mediated by UBE2C and UBE2S in the cell cycle are vital for the orderly and accurate progress of the cell cycle [166]. The formation of heterotypic chains with multiple different lysine linkages on CCNB1 is determined by the concentration of UBE2C within the APC/C Ub reaction. Low concentrations of UBE2C form heterotypic chains predominantly containing Lys11/Lys48 linkages, but at high concentrations, UBE2C forms complex chains with six different Ub linkages. UBE2C or UBE2D co-operate with APC/C for the initial ubiquitin attachment to substrates, while UBE2S co-operates with APC/C for subsequent ubiquitin attachments, producing Lys11 chains [167].

In addition, UBE2C, UBE2D2, UBE2D3 [168], UBE2N, UBE2Q1, UBE2S, and UBE2T [169] can affect the stability and activity of p53 protein to mediate cell cycle arrest. p53 acts as a homotetramer transcription factor, regulating the expression of genes including transactivation of its target genes, including *cyclin dependent kinase inhibitor 1A* (CDKN1A, also known as p21/Cip1), *GADD45*, and *stratifin* (SFN, also known as 14-3-3σ) or inhibits *CDC2* and *CCNB1* expression [170]. Mutation of p53 upregulates *CCND1* and downregulates *CDKN1A*, leading to a highly active CDK4/CDK6 complex and thus ensuring uninterrupted pancreatic cancer cell division and uncontrolled cell proliferation [171]. Overexpression of *UBE2S* in HCC functions as an oncogene by increasing the ubiquitination of p53 [172]. HEK293 cells overcame radiation-induced G2/M cell cycle arrest through ubiquitination of p53 via UBE2K. Phosphorylated UBE2K inhibits p53, thereby suppressing the expression of CDKN1A, and sequentially stimulating CCND and CCNE to induce re-entry into the cell cycle. This suggests that UBE2K plays a regulatory role in ultraviolet-induced cell cycle arrest and re-entry [173,174]. Knockdown of *UBE2Q1* reduced HCC cell proliferation, promoted apoptosis via induction of GADD45α, and suppressed orthotopic tumorigenicity both in vitro and in vivo [175].

Disorders of cell cycle progression play a key role in the formation and development of cancer, and the abnormal regulation of the cell cycle often leads to the occurrence and development of cancer. *UBE2S* is highly expressed in melanoma cells and tissues. Treatment of melanoma cells with a short hairpin RNA targeting *UBE2S* led to cell cycle arrest in the G1/S phase and inhibition of cancer cell growth [176]. *UBE2T* knockout can significantly inhibit the proliferation and colony formation of bladder cancer cells, induce cell cycle arrest, and increase the rate of apoptosis, which might be related to the fact that *UBE2T* is the target gene promoted by the E2F transcription factor [177]. Inhibition of *UBE2T* in GC cells arrested the cell cycle in the G2/M phase, and ultimately inhibited cell proliferation and colony formation [178]. UBE2C and APC/C affect the proliferation rate and cell cycle distribution of esophageal cancer cells by interfering with the level of CCNB [179]. The cell cycle regulator FOXM1 binds to the *UBE2C* promoter region in esophageal cancer cells and activates its transcription, leading to upregulation of *UBE2C* expression [180]. Overexpression of UBE2C can also induce epithelial to mesenchyme transition (EMT) related to cancer cell invasion and metastasis through the APC/C complex and Wnt/β-catenin [7], P13K/AKT, and p53 [181] signaling pathways. EMT is closely related to the occurrence and development of cancer. Silencing *UBE2C* causes pancreatic ductal adenocarcinoma cells to block in the G1/S phase, and reduces the levels of EMT markers (VIM, E-cadherin) [182]. E2s that regulate EMT also include UBE2O, UBE2V1, and UBE2T [183]. Flavopiridol can significantly inhibit the activity of CDKs (1/2/4/6/7) by interacting with the ATP binding pocket on the kinase, thereby causing G1/G2/M phase block. It is a classic non-selective CDK inhibitor and has successfully entered clinical trials [184]. Thus, blocking the cell cycle of malignant cell proliferation-associated diseases, such as cancer, will be the basis for developing new drugs for many years. However, it can be seen from the above that UBE2C and several special E2s promote the progress of cell cycle. At the same time, UBE2N and other E2s regulate p53 to regulate cell cycle. Therefore, the regulation of E2s on cell cycle is complex and extensive, which requires further study of the unique role of single E2 in cancer cell.

### 4.3. Apoptosis

The UPP plays a key role in the regulation of apoptosis. Currently, three apoptosis pathways are studied, namely the death receptor-mediated pathway, the mitochondria-mediated pathway, and the endoplasmic reticulum pathway [185]. Regulatory factors of apoptosis include B-cell leukemia/lymphoma 2 protein family (BCL2), inhibitor of apoptosis proteins (IAPs), and IκB kinase (IKK) regulatory factors. Some regulatory molecules involved in apoptosis have been identified as proteasome substrates [186]. BIRC6 is a member of the IAP family. Its overexpression prevented apoptosis by ubiquitinating and degrading second mitochondria-derived activator of caspases (SMAC) and caspase 9 in mouse fibroblasts [187]. BIRC6 can also bind to other active caspases, including caspases 3, 6, and 7, and such interactions have been shown to underlie its ability to inhibit the caspase cascade and ultimately apoptosis [188]. Knockout of *BIRC6* can block the CRC cell cycle in the S phase and increase apoptosis [189]. High levels of BIRC6 protein are related to poor clinical manifestations of patients with prostate cancer (PCa) [190], non-small cell lung cancer (NSCLC) [191], acute leukemia [192], and epithelial OC [35], which are related to poor prognosis. It can be seen that targeting E2 member BIRC6 in cancer cell can release the inhibition and degradation of caspase activity, thus affecting the survival of cancer cell. Inhibition of apoptosis prevents the death of cancer cells, either associated with carcinogenic initiation or cancer therapy [193]. UBE2O-mediated c-Maf ubiquitination and degradation, which can induce multiple myeloma cell apoptosis [194]. UBE2M promoted osteoarthritis chondrocyte apoptosis by activating the Axin-dependent Wnt/β-catenin pathway [195].

Certain E2s, such as UBE2B, UBE2C, UBE2D, UBE2Q, and BIRC6, function to regulate cell apoptosis through the ubiquitination and degradation of p53. p53 regulates apoptosis by regulating genes such as *PUMA*, *NOXA*, *BIM*, *BAX*, *BCL2*, *FAS*, and *IGFBP3* (Figure 4B), and it can also induce cell apoptosis through the death signal receptor protein pathway (TNF receptor and Fas protein) [182]. Suppression of UBE2D can stabilize p53, leading to enhanced apoptosis and markedly inhibited proliferation of human LC cells in a p53-dependent manner [196]. The mutation of *BIRC6* led to upregulation of p53, resulting in mitochondrial apoptosis [197]. The upregulation of UBE2D1 in HCC promoted the growth of HCC, which was achieved by mediating the ubiquitination and degradation of p53 [198]. Complete silencing of *UBE2I* in RAW264.7 cells resulted in apoptosis. Downregulation of UBE2I decreased the level of the BCL2 protein in HepG2 cells and thus caused HCC cell apoptosis [117]. These reports showed that UBE2I is necessary for cancer cell survival. The above studies indicated that multiple E2 members can directly or indirectly regulate the apoptosis of cancer cells. To a large extent, the drug resistance of cancer cells is also based on the resistance to apoptosis. This observation will be useful to develop treatments for diseases such as cancer and abnormal cell proliferation.

### 4.4. The Wnt/β-Catenin Pathway

Wnt/β-catenin signaling pathway plays an important role in embryonic development [199]. As the core component of this pathway, β-catenin is tightly regulated by post-translational modifications that fine-tune its protein level and optimal activity. β-catenin is phosphorylated by CK1 and GSK-3β and then enters the UPP. Under the combined action of SKP1/CUL1/F-box protein complex-β-transducin repeat-containing protein (SCF-βTrCP) and some E2s, β-catenin is degraded, which stabilize the concentration of β-catenin in the cytoplasm and block the Wnt/β-catenin signaling pathway [200]. As a novel activator of the Wnt/β-catenin signaling pathway, UBE2S modifies β-catenin at Lys19 via a Lys11-linked polyUb chain. This modification promotes β-catenin stabilization through antagonizing its Lys48-linked proteasomal degradation, mediated by the destruction complex/βTrCP signaling [201]. UBE2B can also stabilize β-catenin through the Lys63-linked polyUb chain, and silencing *UBE2B* can inhibit the transcription activity of β-catenin [202]. This is because *UBE2B* is the transcription target of β-catenin/T-cell factor. Even if β-catenin is ubiquitinated, it is stable and transcriptionally active, indicating that there is positive feedback regulation between UBE2B and β-catenin [203].

If the activity of E2 or E3 changes, it will destroy the stability of β-catenin. The concentration of β-catenin might increase in the cytoplasm, which in turn activates the Wnt/β-catenin signaling pathway, and ultimately regulates the transcription of target genes *C-MYC*, *CCND1*, and *matrix metalloproteinase 7 (MMP7)* [204]. Overexpression of UBE2S significantly upregulated the expression of β-catenin, CCND1, and MMP7, and the activity of Wnt/β-catenin signaling in A549 cells [205]. In addition, since Wnt/β-catenin signaling is crucial for the activity of epithelial stem cells, it is not surprising that Wnt/β-catenin pathway mutations are frequently observed in carcinomas [206]. The positive regulatory relationship between UBE2S and hypoxia-inducible factor 1α (HIF-1α) in cancer cells might contribute to greater proliferation, angiogenesis, and metastasis of cancer [207]. Overexpression of UBE2S can also activate β-catenin signaling by downregulating SOX6, inhibiting the activity of CCNB1, and promoting the proliferation and migration of endometrial cancer cells [208]. UBE2T downregulation suppressed the activity of the Wnt/β-catenin signaling pathway, which ultimately suppressed NSCLC cell proliferation, migration, and invasion in vitro [209]. The pathway activation and in vitro pro-metastasis effects of UBE2T were blocked using an AKT inhibitor. Therefore, UBE2T might promote the development and progression of NPC by activating the AKT/GSK3β/β-catenin pathway [210]. However, it remains challenging to develop effective molecules that specifically target this pathway to improve cancer therapy. We believe that maintaining the activity and expression level of related E2s in cancer cells might be a way to control cancer cell proliferation and metastasis.

### 4.5. Nuclear Factor-Kappa B (NF-κB) Pathway

**Figure 5:**
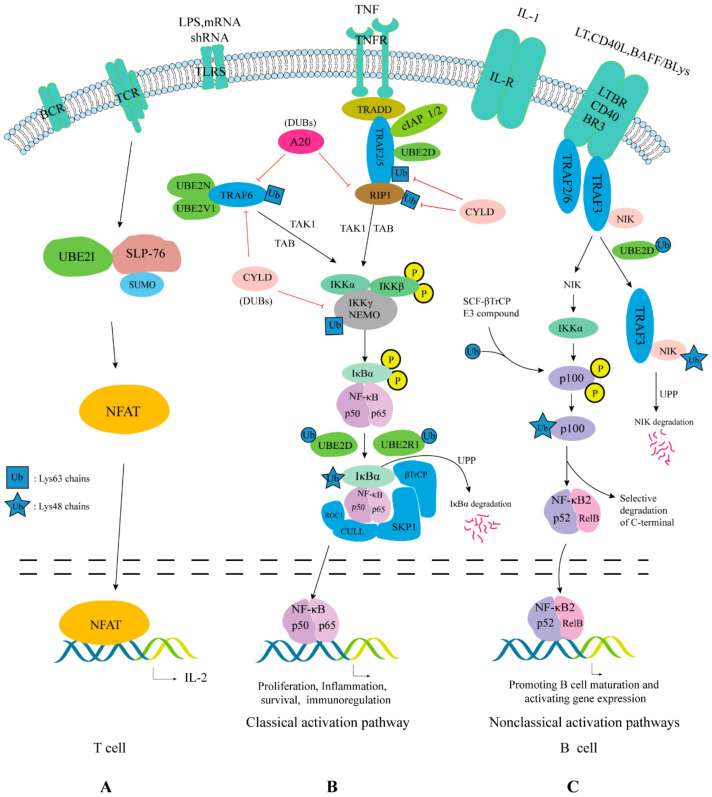
E2s participate in the NF-κB pathway. (**A**) T cell receptor (TCR) stimulation promotes the binding of SLP-76 and UBE2I and increases the SUMOylation of SLP-76. SLP-76 and UBE2I synergize to augment TCR-mediated *IL2* transcription by nuclear factor of activated T cells (NFAT) in a manner dependent on SUMOylation of SLP-76 [232]. (**B**) Under the action of the stimulation signal, IκBα will rapidly undergo phosphorylation modification under the stimulation of IKK. IKK contains two catalytic subunits, IKKα and IKKβ, and one regulatory subunit IKKγ [225]. In the classical pathway, IKKβ phosphorylates IκBα and is then recruited into the SKP1/CUL1/F-box protein (SCF) complex via βTrCP containing the F-box domain. βTrCP contains a WD40-repeat domain, which can specifically bind to two phosphorylated serine sites on the N-terminus of IκBα [233]. The RING domain contained in ROC1 can recruit E2 and to polyubiquitinylate phosphorylated IκBα, so that it is degraded by the 26S proteasome and “releases” NF-κB into the nucleus [234]. (**C**) In the non-classical pathway, activation of the pathway was suggested to depend on receptor-induced TRAF3 degradation and stabilization of NF-κB inducing kinase (NIK) [235]. TRAF3 physically associates with NIK via a specific sequence motif located in the N-region of NIK and ubiquitinates NIK to stabilize it with UBE2D [226]. CD40 and B cell activativing factor belonging to the TNF family (BAFF) of the tumor necrosis factor (TNFR) superfamily activate NIK selectively, and then NIK phosphorylates and activates IKKα. Activated IKKα catalyzes the phosphorylation of the two Ser sites at the C-terminus of the p100 precursor protein, and the phosphorylated modified p100 is recognized by the SCF-βTrCP complex [236]. The proteasome only degrades the C-terminus of p100 that contains ankyrin repeats, but does not affect the N-terminus (that is, the p52 subunit) containing the rel-homology domain (RHD) [237]. Then, p52 combines with RelB to form a dimer, which promotes the expression of target genes that can mature and activate B cells [238].

E2s participate in signaling pathways by modifying certain key substrates, including mTORC1, PTEN-AKT, TNF, TLR, NLR, RLR, and TCR. In addition to acting through NF-κB, it also acts through interferon regulatory factors (This topic has recently been expertly reviewed [211]). The NF-κB signaling system (including the NF-κB dimer, IκB, and IKK) can respond to stimuli from a variety of signaling pathways such as TNF, TLR, NLR, RLR, and TCR [212]. Although NF-κB exists widely, it exists in an inactive state in most cells. This is because NF-κB is “trapped” by IκB in the cytoplasm of unstimulated cells [213]. The degradation of IκB, the key inhibitor of NF-κB, is coordinated by at least four post-translational modifications, allowing activation only when several prerequisites are met [165]. Under the action of various stimulating factors, including microorganisms, UBE2N and UBE2V1 form a heterodimer, which binds to the loop domain of TNF receptor associated factor (TRAF) family of adaptor proteins. This promotes the activation of TNFR and TLR protein kinases involved in signal transmission [214]. UBE2D1 can collaborate with c-IAP1 and mediate TNF-α stimulated receptor-interacting protein 1 (RIP1) ubiquitination [215]. UBE2N-UBE2V1, TRAF6, and TRAF2 are involved in the formation of Lys63-linked chain on NF-κB essential modifier (NEMO)/IKKγ and/or RIP1 [216,217,218,219]. After a series of reactions, IκB is phosphorylated and activated. After phosphorylated IκB enters the UPP, it forms a Lys48-linked ubiquitin chain under a two-step mechanism mediated by UBE2D and UBE2R1 [220]. Finally, IκB is rapidly degraded, releasing NF-κB into the nucleus, where it activates the transcription of a series of genes [221] (Figure 5). UBE2N-UBE2V1 not only mediates NF-κB activation, but also activates the RHBDF2-TACE signaling pathway to antagonize NF-κB activation. Thus, UBE2N-UBE2V1 plays dual roles in regulating the NF-κB pathway [222]. Besides, UBE2I also has a dual role in the regulation of NF-κB. SUMO1 acylates IκB and inhibits its ubiquitination, while SUMO2/3 plays the opposite role, that is, it promotes the ubiquitination and degradation of IκB [223]. The products of NF-κB target genes *TNFAIP* (TNF alpha induced protein 3, also known as A20) and *IκBα* terminate NF-κB activation.

In normal cells, NF-κB becomes activated only after the appropriate stimuli, and then upregulates the transcription of its target genes [224]. In cancer cells, different types of molecular alterations might result in impaired regulation of NF-κB activation. In such case, NF-κB loses its inducibility and becomes constitutively activated [225]. This leads to deregulated expression of genes under NF-κB control. Although NF-κB is known to regulate immune and inflammatory responses, it also regulates the expression of cancer cell related genes such as those involved in proliferation (e.g., *CCND1*), angiogenesis (e.g., *VEGF*), invasion (e.g., *MMP9)*, metastasis (e.g., *ICAM1*), and apoptosis (Promoting apoptosis (e.g., *FAS*) or Inhibiting apoptosis (e.g., *BCL2*)) [226]. *UBE2V1* mRNA levels appeared to be elevated in all cancer cell lines examined [227]. Constitutive high-level expression of UBE2V1 alone in cultured human cells was sufficient to cause a significant increase in NF-κB activity, as well as the expression of its target anti-apoptotic protein, BCL2 [228]. Overexpression of UBE2V1 can inhibit stress-induced apoptosis of HepG2 cells by activating NF-κB signaling. Overexpression of UBE2V1 alone in BC cells was sufficient to activate NF-κB, which in turn upregulated the MMP1 expression to enhance BC cell metastasis [229]. Collectively, these observations establish a close correlation between UBE2V1 expression and tumorigenic potential. The N-terminal region of UBE2V1 is the molecular determinant of its cellular functions in the NF-κB signaling pathway. Therefore, an ideal inhibitor should target the N-terminal region of UBE2V1 instead of the UBE2N-UBE2V1 interface. RIP1 forms a Lys11-linked polyUb chain under the action of UBE2D, UBE2S, and c-IAP1. NEMO effectively binds to the Lys11-linked polyUb chain, which might play a signal role in the activation of cell proliferation pathways [230]. After NF-κB is activated, it can stimulate activated macrophages to release pro-inflammatory mediators and promote the growth of cancer cells [231]. These imply a tumor promotion function of E2.

### 4.6. Other Cases

The number of identified substrates conjugated by a particular Ub varies considerably, with tens of thousands of substrates identified for ubiquitin, thousands for SUMO, just a few for URM1, and two for ATG [32]. Therefore, there are many more E2s modified substrates that could participate in the regulation of cancer. For example, the UBE2N-UE2V1A complex and TRAF6 together trigger the Lys63 ubiquitination of AKT kinase. Then, AKT is phosphorylated to inhibit the regulation of BC cell cycle arrest and apoptosis by the FOXO1 transcription factor [239]. UBE2N activates non-SMAD signals through the TAK1-p38 MAP kinase cascade to regulate BC transfer, which is achieved by UBE2N activation of mitogen-activated protein/ERK kinase kinase 1 (MEKK1) and TAK1 [11]. UBE2T promoted the proliferation of renal cell carcinoma cells by regulating P13K/AKT signaling, suggesting that it might be a novel target for the treatment of patients with renal cell carcinoma [240]. UBE2N and UBE2D are required to initiate monoubiquitination and subsequent polyubiquitination of MHC class I molecules, which is necessary for their efficient endocytosis and endolysosomal degradation [241]. The UBE2D complex is critical to maintain KRAS protein stability, and targeting such a complex might be a unique strategy to degrade mutant KRAS to kill cancer cells [242]. Downregulation of UBE2O promoted AMPKα2-mediated suppression of the (mTORC1)-HIF-1α pathway, which is essential for metabolic “reprogramming” of cancer cells [243]. SUMOylated collapsin response mediator protein 2 (CRMP2) exists widely in GBM cells. Inhibition of CRMP2 SUMOylation could suppress GBM proliferation significantly in vitro [244].

Although cancers are derived from numerous tissues with multiple etiologies, and its progression carries with bewildering and seemingly endless combination of genetic and epigenetic alterations. However, there is a relatively small amount of “mission critical” events: deregulated cell proliferation and suppressed apoptosis. E2s not only promote the overexpression of these “mission critical” events in cancer cells, but also promote the progress of their entire life cycle. Understanding the biological processes involved and the related cancers are necessary for the initial selection of anti-cancer targets (Table 1).

## 5. Inhibitors and miRNAs Targeting E2s

Cancer cells’ rapid division and disordered regulatory pathways make them more susceptible to proteasome inhibition; therefore, proteasome inhibitors have become a new class of chemotherapeutic drugs that cause cell cycle arrest and cell death. We know that the UPP is currently one of the most widely studied target in disease treatment. Velcade and Krypolis, which inhibit the activity of the 26S proteasome, have been developed successfully to treat a variety of blood diseases [245]. The proteasome is the last step of the ubiquitination process and targets large amounts of ubiquitinated proteins for degradation; therefore, inhibiting the proteasome might lead to the accumulation of upstream ubiquitinated proteins. This could have serious consequences, which limit the widespread use of these drugs. Therefore, we could turn our attention to the specific components in the UPP, which might be a better strategy to specifically suppress them. Currently, inhibitors targeting E3 ligases and DUBs have been used in clinical trials to treat cancer [246]. Inhibitors targeting E2s are still in basic research, such as preliminary screening. There are few E1s; therefore, targeting E1s might affect multiple ubiquitination pathways. Based on the central position of E2s in the UPP and their appropriate quantity and special structure, one or more of E2s could be considered as targets to develop anticancer drugs, so as to provide the specificity that E1 inhibitors cannot achieve.

Most of the E2 inhibitors currently studied change activity of E2s by binding to the active site, allosteric site, or protein interaction site (Table 2). UBE2I is an anti-cancer target for cancers driven by MYC and RAS/RAF. Researchers used the X-ray crystal fragment screening method to find an allosteric small molecule binding site on the back of UBE2I. They believe that compounds that bind to this site might interfere with various protein-protein interactions in UBE2I’s activities [247]. It was reported that arsenic can cross-link adjacent cysteines in the catalytic domain of UBE2O, which can be used as a method to inhibit UBE2O activity. Arsenic is currently in clinical trials for various cancers [30]. The ligand molecules of the UBE2D2 activate site obtained through virtual screening include pyridine, piperidine, and azol ring scaffolds, which can be used as candidates for the development of anticancer drugs [248].

In addition to directly targeting the structure of E2s using small molecule inhibitors, the use of miRNAs to inhibit E2 mRNAs might also affect the protein turnover process of cancer cells. miRNAs are endogenous small noncoding RNAs that participate in the regulation of gene expression [267]. miRNAs, as natural antisense nucleotides, showed reduced immune response and low toxicity compared with plasmid DNA-based gene therapy and protein-based drug molecules [268]. However, the low stability of miRNAs and their difficult delivery into cells restrict their application in clinical practice. The delivery of miRNAs combined into nanostructures might improve their biodistribution and accumulation at the target site, with some papers showing encouraging results, both in in vitro and in vivo [269]. miRNA drugs can target molecules that cannot be targeted by chemical drugs or antibody drugs, and are expected to make breakthroughs in cancers with poor efficacy under traditional drugs treatment. Table 2 summarizes the miRNAs that target E2 members, providing a reference for the development of miRNA anticancer drugs.

## 6. Concluding Remarks and Future Perspectives

Through this review, we can see that E2s participate in different biological processes by modifying different molecules, and their abnormal activation or dysfunction might lead to the occurrence and development of cancer. Targeting certain E2 alone might affect a variety of biological processes. For example, the inhibition of UBE2N might affect the NF-κB pathway involving UBE2V1 and the DNA repair pathway involving UBE2V2. IJ-5 exerts an anti-inflammatory effect by binding to the active cysteine site of UBE2D; however, enzymes such as DUBs, other cysteine proteases, and protein tyrosine phosphatases also have cysteine active sites. Thus, IJ-5 might also inactivate these enzymes.

Based on a wide range of substrates and functions that are affected by E2s, it is necessary to use appropriate in vivo models and genetic methods to study the unique mode of action, functions, and specific modifications performed by a single E2 in different biological environments. Finally, new treatment options will be studied for a single E2, E2 complex, or specific E2-E3 interaction. In addition, it is necessary to understand the relationship between the principle of a drug’s action and the pathogenesis of the treated disease, and to develop the best drugs for comprehensive clinical evaluation. The development of drugs is not done overnight. Although some compounds that inhibit E2s are summarized in Table 2, they are limited to experimental studies. Therefore, the development of E2 inhibitors for clinical treatment remains challenging. Specific modifications to the reported E2 inhibitors to reduce these shortcomings, optimize their efficacy, and their use in combined applications (chemotherapy drugs and inhibitors or multi-target inhibitors), might achieve unexpected results.

This article clarifies the molecular basis of E2s as a cancer treatment target and the efforts made to develop E2 inhibitors based on the special position and role of E2s in the UPP, the structure of E2s, and the biological processes that E2s participate in. Currently, no E2 inhibitor is actually used in the clinical treatment of cancer; however, its potential as a therapeutic target cannot be ignored. In addition, this review also provides a deeper understanding of the various roles played by E2s in cells and the impact it produces, which could provide a reference for basic research into E2s.

## Figures and Tables

**Table 1:** E2s, its synonyms, classification, relevant biological roles, and involvement in cancers.

Name (Human)	Synonyms	Classification	Biological Roles	Relevant Cancers
UBE2A	RAD6(A)	Class I	Transcriptional regulation	Chronic myeloid leukemia
			DNA repair [33]	
			Regulating myeloid differentiation [34]	
UBE2B	RAD6(B)	Class I	Ubiquitinating H2A/B and MGMT to participate in DNA repair	MM, BC
			Monoubiquitinating H2B to participate in transcriptional activation	
UBE2C	UBCH10	Class II	Ubiquitinating p53 and Ki67 [35] to participate in G2/M transition [36]	MM of uterus, Melanoma, HCC [37], HNSCC [38], CRC [39], Glioma [40], TSCC [41], Cerebral cancer, LC, Leukemia, Lymphoma, GC [6,7,8,9], BC, Esophageal cancer, CC, Endometrial carcinoma, OC
			Regulating the level of phosphorylated ERK1/2 to participate in cell apoptosis [42]	
			Its depletion reduced OC malignancy and reversed DDP resistance via downregulating CDK1 [43]	
UBE2D(1/2/3)	UBCH5(a/b/c)	Class I	Regulating the level of p53 protein [44]	Esophageal cancer [45], PCa
			Ubiquitinating MDM2 and CCND1	
UBE2E1	UBCH6	Class II	Affects the patient’s response to induction chemotherapy [46]	Acute myelogenous leukemia, PCa
UBE2E2	UBCH8	Class II	Stable substrate protein with ISG15 can promote cancer cell movement and invasion [47]	BC
UBE2E3	UBCH9	Class II	Maintaining mitochondrial homeostasis [48], participating in NEDD4^−^ dependent epithelial Na^+^ channel regulation [49]	-
UBE2G1	UBE2G/E217K	Class I	Regulating inflammation and innate immune response [50], ubiquitinating and degrading of IKZF1 and IKZF3 [51]	Myeloma
UBE2G2	UBC7	Class I	Co-regulating immune receptor downregulation mediated by human cytomegalovirus US2 with TRC8 [52]	NSCLC [53], PCa [54]
UBE2H	UBC8/UBCH2	Class III	Participating in neurodevelopment [55]	-
			TNF-α promotes the binding of the *UBE2H* promoter region to NF-κB [56]	
UBE2J1	UBC6	Class III	It negatively regulates interferon to promote RNA virus infection [57]	Medulloblastoma [58], PCa
			Participating in spermatogenesis and growth and development	
UBE2J2	NCUBE2	Class III	Regulates ERAD induced by human cytomegalovirus US2 through TRC8 [52]	HCC [59]
UBE2K	UBCH1/E2-25k	Class III	Regulating the cell cycle	-
UBE2L3	UBCH7	Class I	Participating in DSB repair	HCC [60], Cervical Cancer [61], NSCLC [62], B-cell lymphoma
			Ubiquitinating p53 and p27^Kip1^ to regulate the cell cycle [63]	
			Regulating the NF-κB signal driven by TNF-α [64], Rate limiting factors and therapeutic targets of LUBAC activity [65]	
UBE2N	UBC13	Class I	UBE2N-UBE2V1 complex regulates innate immunity and participates in the activation of NF-κB [66]	BC, Cervical Cancer [67], HCC [68], DLBCL, LC, Malignant melanoma [69]
			UBE2N-UBE2V2 ubiquitinates PCNA and H2A	
			Ubiquitinating and degrading Sirt1 and inhibiting histone H4 lysine 16 acetylation [70]	
			Activating MAPKs	
			Involved in the internalization of cell surface receptors	
UBE2O	E2-230K	Class IV	Ubiquitinates BMAL1 to regulate transcriptional activity and circadian rhythm function [71]	BC [72], GC, RC [5], Anemia, MM, OC, HNSCC [73], LC [74]
			Participating in erythropoiesis, ubiquitinating RPs to participate terminal erythroid differentiation [75]	
			Regulating apoptosis	
			Monoubiquitinating SMAD6 to participate in bone morphogenesis [76]	
			Ubiquitinating and degrading MXI1 at the Lys46 residue	
UBE2Q1	UBE2Q/NICE5	Class II	Regulating p53 [77]	HCC, BC, ALL [78], CRC [79]
			Regulation of lysosome integrity and lysophagy [80]	
UBE2Q2	Nothing	Class II	Regulating apoptosis	HNSCC [81], CRC [82]
UBE2R1	CDC34/UBC3/UBCH3	Class III	Ubiquitinating and degrading p27^Kip1^ [83] and IκBα	MM, HCC [84], NSCLC [85], ALL [86]
UBE2S	E2EPF/EPF5	Class III	Ubiquitinating CDKN1A, CCNB1, CDC20, and p53 (Lys11/Lys48 polyUb chain) to regulate apoptosis	HCC [68], BC, OSCC [87], NSCLC [88], Melanoma [89], CRC
			Ubiquitinating β-catenin to maintain its stability	
			Ubiquitinating SOX2 to regulate neuroectodermal differentiation and maintaining mES cells [90]	
UBE2T	FANCT/PIG50/	Class III	Nucleic acid excision repair for UV damage [91]	FA [65,92], GC [93], Osteosarcoma [94], PCa [95], LC [96], BC [97], HCC [98], Intrahepatic cholangiocarcinoma [99], Gallbladder cancer [100], NPC, CRC, MM [101], OC [102]
			Ubiquitinating and degrading p53 [101]	
			Participating in Wnt/β-catenin signaling and P13K/AKT signaling, regulating BRCA1 degradation	
UBE2V1	UEV1A	Class II	It participates in the activation of NF-κB together with UBE2N [71]	Metastatic CRC, BC, Osteosarcoma [103]
UBE2V2	MMS2	Class I	Participates in DNA repair together with UBE2N	-
UBE2W	UBC16	Class I	UBE2W downregulation promotes cell apoptosis and correlates with hypospermatogenesis [104]	-
BIRC6	Appolon/BRUCE	Class IV	A positive regulator of macroautophagy/autophagy [105]	HCC [106,107], NB [35,108], CRC, PCa [109], OC [35]
**Ub-like**				
UBE2F	NCE2	Class II	Promoting the survival of lung cancer cells [110]	LC
UBE2I	UBC9	Class I	Promoting the development of T cells [111], SUMOylation of IRF4 promotes the M2 process of macrophages [112], SUMOylation of IRF7 limits its transcriptional activity [113]	HCC [114], BC
			SUMOylation of (SUMO1) NLRP3 activates the inflammasome, Regulating the NF-κB signaling [115]	
			Participating in the formation of Lys49 polyUb chain to resist senescence [116]	
			Affects BCL2 expression through the ER signaling pathway [117]	
			UBE2I-PCGF2 complex inhibits the SUMOylation of PML-RARA [118]	
			Participating in the development and survival of CLPs [119]	
UBE2M	UBC12/UBC-RS2	Class II	DNA repair [120]	HCC [121], RC [122], LC, Intrahepatic cholangiocarcinoma [123]
			Ubiquitinating and degrading UBE2F [124]	
			Participating in the cell cycle [125]	
UBE2Z	USE1/HOYS7	Class IV	Participating in the ERK and STAT3 signal pathway [126]	HCC

**Table 2:** Inhibitors and miRNAs targeting E2s.

Name	Target	Origin	Inhibition Mechanisms	Test Diseases	Characteristics
**Inhibitors**					
IJ-5 [249]	UBE2D3	Herb	Combines with Cys85 of UBE2D3 to inhibit NF-κB signaling	Arthritis, Hepatitis	Difficulty in synthesis
Compound 6d [250]	UBE2D3	α-Santonin derivatives	Same as above	Arthritis	The efficacy of 6d is greater than IJ-5, but 6d is unstable
1β-hydroxy alantolactone [251]	UBE2D	Herbal medicine	Same as above	Inflammation	It is more efficient in combination with UBE2D3
CW3 [252]	UBE2G2	Synthesis	The vinyl group of CW3 inhibits E2 by forming a covalent bond with the thiol group of Cys48 of UBE2G2	Melanoma	-
TZ9 [253]	UBE2B	Synthesis	-	BC	Selective suppression
New triazine drugs (6a-c) [151]	UBE2B	Based on TZ9 synthesis	It incorporates deep inside the UBE2B binding pocket by interaction with UBE2B active site residues Cys88 and Asp90.	OC, LC, BC, CC	Inhibitory activity > TZ9, Selective suppression
CC0651 [254]	UBE2R1	Synthesis	It inserts into the hidden binding pocket of the non-catalytic site of UBE2R1 and interferes with the release of Ub to the Lys residue of the substrate	-	Allosteric inhibition
2-D08 [255]	UBE2I	Synthesis	Preventing transfer of SUMO from the UBE2I-SUMO thioester to the substrate	-	In vitro biochemical test
Compound 2 [256]	UBE2I	Synthesis	Binding near the active site of UBE2I	-	Low potency, low selectivity
Leucettamol A [257]	UBE2N	*Leucetta aff. microrhaphis*	Inhibiting the formation of the UBE2N-UBE2V1 complex	-	Its hydrogenation increased its inhibitory activity
Manadosterols A and B [258]	UBE2N	*Lissodendoryx fibrosa manadosterols*	Same as above	-	The activities are more potent than those of Leucettamol A
NSC697923 [259]	UBE2N	Synthesis	Impeding the formation of the UBE2N and Ub thioester conjugate.	NB	Efficacy > Doxorubicin and Etoposide
Luteolin and Quercetin [260]	UBE2S	Plants	-	Cervical cancer	-
CU2 [261]	UBE2T	Synthesis	Inhibiting UBE2T/FANCL-mediated FANCD2 monoubiquitylation	-	Cell and biochemical tests
**miRNAs**					
miR-548e-5p [262]	*UBE2C*	Human LC organization	Binding to the 3′-UTR of *UBE2C*	NSCLC	In vitro test
miR661-3p [263]	*UBE2C*	Human 293 cells	Binding to the 3′-UTR of *UBE2C*	NSCLC	In vivo and in vitro tests
miR-381-3p [264]	*UBE2C*	Human PCa cells	ICT upregulates the level of miR-381-3p to downregulate the expression of *UBE2C* in human PCa cells	PCa	In vivo and in vitro tests
miR-147b [69]	*UBE2N*	HC organization	Binding to 3′-UTR of *UBE2N*	HC	In vivo and in vitro tests
miR-1305 [265]	*UBE2T*	Synthesis	Binding to 3′-UTR of *UBE2T*	LC	In vivo and in vitro tests
miR-214 [266]	*UBE2I*	Synthesis	Binding to 3′-UTR of *UBE2I*	Glioma	In vitro test

## Data Availability

Not applicable.

## Abbreviations

ALL	Acute Lymphoblastic Leukemia
BC	Breast cancer
BMAL1	Brain and muscle arnt-like 1
C-	C-terminal
CCN (A/B/D/E)	Cell cyclin (A/B/D/E)
CDK	Cyclin-dependent kinase
CLPs	Common lymphatic progenitor cells
CRLs	Cullin-RING ligases
DLBCL	Diffuse large B-cell lymphoma
DUB	Deubiquitinating enzyme
E2	Ubiquitin-conjugating enzyme
EBV	Epstein-Barr virus
ERAD	Endoplasmic reticulum-associated degradation
FA	Fanconi
GBM	Glioblastoma
HCC	Hepatocellular carcinoma
HR	Homologous recombination
ICL	Interstrand cross-link
IKK	IκB kinase
LC	Lung cancer
LUBAC	Linear ubiquitin chain assembly complex
mES	Mouse embryonic stem cells
MM	Multiple myeloma
MMP	Matrix metalloprotinase
N-	N-terminal
NF-κB	Nuclear factor-kappa B
NIK	NF-κB inducing kinase
NSCLC	Non-small cell lung cancer
OSCC	Oral squamous cell carcinoma
PCa	Prostate cancer
PCNA	Proliferating cell nuclear antigen
polyUb	Polyubiquitin
RING	Really interesting new gene
RPs	Ribosomal proteins
SCF	SKP1/CUL1/F-box protein complex
TLS	Translesion synthesis
TNF-α	Tumor necrosis factor α
βTrCP	β-transducin repeat-containing protein
Ub	Ubiquitin
Ubl	Ub-like
APC/C	Anaphase-promoting complex/cyclosome
BCL2	B-cell leukemia/lymphoma 2
BRCA1	Breast cancer 1
CC	Colon cancer
CDC20	Cell division cycle 20
CDKN1A/p21/Cip1	Cyclin dependent kinase inhibitor 1A
CRC	Colorectal cancer
DDP	Cisplatin
DSBR	DNA Double strand break repair
E1	Ubiquitin activating enzyme
E3	Ubiquitin ligase
EMT	Epithelial to mesenchyme transition
ERK	Extracellular regulated protein kinases
FOXM1	Forkhead box protein M1
GC	Gastric cancer
HNSCC	Head and neck squamous cell carcinoma
IAPs	Inhibitor of apoptosis proteins
ICT	Icaritin
IκB	Inhibitor of nuclear factor kappa B
LCSCs	Liver cancer stem cell
MDM2	Mouse double minute 2 homolog
miRNAs	microRNAs
MMC	Mitomycin C
MXI1	Max-interacting protein 1
NB	Neuroblastoma
NHEJ	Non-homologous end joining
NPC	Nasopharyngeal carcinoma
OC	Ovarian cancer
p27^Kip1^	Kinase inhibition protein p27
PCGF2	Polycomb group ring finger 2
PML-RARA	Promyelocytic leukemia (PML) and the retinoic acid receptor-α (RARA)
RC	Renal carcinoma
RIP1	Receptor-interacting protein 1
SAC	Spindle-assembly checkpoint
SUMO	Small ubiquitin-like modifier
TNBC	Triple negative breast cancer
TRAF	TNF receptor associated factor
TSCC	Tongue Squamous Cell Carcinoma
UBC	Ubiquitin-conjugating
UPP	Ubiquitin-Proteasome Pathway

## References

[1] Wilkinson K.D. (2005). The discovery of ubiquitin-dependent proteolysis. Proc. Natl. Acad. Sci. USA.

[2] Hicke L., Dunn R. (2003). Regulation of membrane protein transport by ubiquitin and ubiquitin-binding proteins. Annu. Rev. Cell Dev. Biol..

[3] Della Sala G., Agriesti F., Mazzoccoli C., Tataranni T., Costantino V., Piccoli C. (2018). Clogging the Ubiquitin-Proteasome Machinery with Marine Natural Products: Last Decade Update. Mar. Drugs.

[4] Ye Y., Rape M. (2009). Building ubiquitin chains: E2 enzymes at work. Nat. Rev. Mol. Cell Biol..

[5] Ullah K., Zubia E., Narayan M., Yang J., Xu G. (2019). Diverse roles of the E2/E3 hybrid enzyme UBE2O in the regulation of protein ubiquitination, cellular functions, and disease onset. FEBS J..

[6] Xie C., Powell C., Yao M., Wu J., Dong Q. (2014). Ubiquitin-conjugating enzyme E2C: A potential cancer biomarker. Int. J. Biochem. Cell Biol..

[7] Wang R., Song Y., Liu X., Wang Q., Wang Y., Li L., Kang C., Zhang Q. (2017). UBE2C induces EMT through Wnt/β-catenin and PI3K/Akt signaling pathways by regulating phosphorylation levels of Aurora-A. Int. J. Oncol..

[8] Zhang H.Q., Zhao G., Ke B., Ma G., Liu G.L., Liang H., Liu L.R., Hao X.S. (2018). Overexpression of UBE2C correlates with poor prognosis in gastric cancer patients. Eur. Rev. Med. Pharmacol. Sci..

[9] Yang M., Qu Y., Shi R., Wu X., Su C., Hu Z., Chang Q., Liu S., Pan G., Lei M. (2016). Ubiquitin-conjugating enzyme UbcH10 promotes gastric cancer growth and is a potential biomarker for gastric cancer. Oncol. Rep..

[10] Van Ree J.H., Jeganathan K.B., Malureanu L., van Deursen J.M. (2010). Overexpression of the E2 ubiquitin-conjugating enzyme UbcH10 causes chromosome missegregation and tumor formation. J. Cell Biol..

[11] Wu X., Zhang W., Font-Burgada J., Palmer T., Hamil A.S., Biswas S.K., Poidinger M., Borcherding N., Xie Q., Ellies L.G. (2014). Ubiquitin-conjugating enzyme Ubc13 controls breast cancer metastasis through a TAK1-p38 MAP kinase cascade. Proc. Natl. Acad. Sci. USA.

[12] Zhang X., Linder S., Bazzaro M. (2020). Drug Development Targeting the Ubiquitin-Proteasome System (UPS) for the Treatment of Human Cancers. Cancers.

[13] Liu W., Tang X., Qi X., Fu X., Ghimire S., Ma R., Li S., Zhang N., Si H. (2020). The Ubiquitin Conjugating Enzyme: An Important Ubiquitin Transfer Platform in Ubiquitin-Proteasome System. Int. J. Mol. Sci.

[14] Xu F.Q., Xue H.W. (2019). The ubiquitin-proteasome system in plant responses to environments. Plant. Cell Environ..

[15] Sadanandom A., Bailey M., Ewan R., Lee J., Nelis S. (2012). The ubiquitin-proteasome system: Central modifier of plant signalling. New Phytol..

[16] Nandi D., Tahiliani P., Kumar A., Chandu D. (2006). The ubiquitin-proteasome system. J. Biosci..

[17] McGinty R.K., Henrici R.C., Tan S. (2014). Crystal structure of the PRC1 ubiquitylation module bound to the nucleosome. Nature.

[18] Pruneda J.N., Smith F.D., Daurie A., Swaney D.L., Villén J., Scott J.D., Stadnyk A.W., Le Trong I., Stenkamp R.E., Klevit R.E. (2014). E2~Ub conjugates regulate the kinase activity of Shigella effector OspG during pathogenesis. EMBO J..

[19] Spit M., Rieser E., Walczak H. (2019). Linear ubiquitination at a glance. J. Cell Sci..

[20] Grice G.L., Lobb I.T., Weekes M.P., Gygi S.P., Antrobus R., Nathan J.A. (2015). The Proteasome Distinguishes between Heterotypic and Homotypic Lysine-11-Linked Polyubiquitin Chains. Cell Rep..

[21] Damgaard R.B., Walker J.A., Marco-Casanova P., Morgan N.V., Titheradge H.L., Elliott P.R., McHale D., Maher E.R., McKenzie A.N.J., Komander D. (2016). The Deubiquitinase OTULIN Is an Essential Negative Regulator of Inflammation and Autoimmunity. Cell.

[22] Meyer H.J., Rape M. (2014). Enhanced protein degradation by branched ubiquitin chains. Cell.

[23] Haglund K., Dikic I. (2005). Ubiquitylation and cell signaling. EMBO J..

[24] Kuang P., Tan M., Zhou W., Zhang Q., Sun Y. (2016). SAG/RBX2 E3 ligase complexes with UBCH10 and UBE2S E2s to ubiquitylate β-TrCP1 via K11-linkage for degradation. Sci. Rep..

[25] Li X., Elmira E., Rohondia S., Wang J., Liu J., Dou Q.P. (2018). A patent review of the ubiquitin ligase system: 2015–2018. Expert Opin. Ther. Pat..

[26] Van Wijk S.J., Timmers H.T. (2010). The family of ubiquitin-conjugating enzymes (E2s): Deciding between life and death of proteins. FASEB J..

[27] Stewart M.D., Ritterhoff T., Klevit R.E., Brzovic P.S. (2016). E2 enzymes: More than just middle men. Cell Res..

[28] Sancho E., Vilá M.R., Sánchez-Pulido L., Lozano J.J., Paciucci R., Nadal M., Fox M., Harvey C., Bercovich B., Loukili N. (1998). Role of UEV-1, an inactive variant of the E2 ubiquitin-conjugating enzymes, in in vitro differentiation and cell cycle behavior of HT-29-M6 intestinal mucosecretory cells. Mol. Cell Biol..

[29] Wenzel D.M., Stoll K.E., Klevit R.E. (2011). E2s: Structurally economical and functionally replete. Biochem. J..

[30] Hormaechea-Agulla D., Kim Y., Song M.S., Song S.J. (2018). New Insights into the Role of E2s in the Pathogenesis of Diseases: Lessons Learned from UBE2O. Mol. Cells.

[31] Garg P., Ceccarelli D.F., Keszei A.F.A., Kurinov I., Sicheri F., Sidhu S.S. (2020). Structural and Functional Analysis of Ubiquitin-based Inhibitors That Target the Backsides of E2 Enzymes. J. Mol. Biol..

[32] Cappadocia L., Lima C.D. (2018). Ubiquitin-like Protein Conjugation: Structures, Chemistry, and Mechanism. Chem. Rev..

[33] Hosseini S.M., Okoye I., Chaleshtari M.G., Hazhirkarzar B., Mohamadnejad J., Azizi G., Hojjat-Farsangi M., Mohammadi H., Shotorbani S.S., Jadidi-Niaragh F. (2019). E2 ubiquitin-conjugating enzymes in cancer: Implications for immunotherapeutic interventions. Clin. Chim. Acta.

[34] Magistroni V., Mauri M., D’Aliberti D., Mezzatesta C., Crespiatico I., Nava M., Fontana D., Sharma N., Parker W., Schreiber A. (2019). De novo *UBE2A* mutations are recurrently acquired during chronic myeloid leukemia progression and interfere with myeloid differentiation pathways. Haematologica.

[35] Wang L., Chen Y.J., Hou J., Wang Y.Y., Tang W.Q., Shen X.Z., Tu R.Q. (2014). Expression and clinical significance of BIRC6 in human epithelial ovarian cancer. Tumour Biol..

[36] Liu G., Zhao J., Pan B., Ma G., Liu L. (2019). UBE2C overexpression in melanoma and its essential role in G2/M transition. J. Cancer.

[37] Wei Z., Liu Y., Qiao S., Li X., Li Q., Zhao J., Hu J., Wei Z., Shan A., Sun X. (2019). Identification of the potential therapeutic target gene *UBE2C* in human hepatocellular carcinoma: An investigation based on GEO and TCGA databases. Oncol. Lett..

[38] Jin Z., Zhao X., Cui L., Xu X., Zhao Y., Younai F., Messadi D., Hu S. (2020). UBE2C promotes the progression of head and neck squamous cell carcinoma. Biochem. Biophys. Res. Commun..

[39] Cacciola N.A., Calabrese C., Malapelle U., Pellino G., De Stefano A., Sepe R., Sgariglia R., Quintavalle C., Federico A., Bianco A. (2016). UbcH10 expression can predict prognosis and sensitivity to the antineoplastic treatment for colorectal cancer patients. Mol. Carcinog..

[40] Ma R., Kang X., Zhang G., Fang F., Du Y., Lv H. (2016). High expression of UBE2C is associated with the aggressive progression and poor outcome of malignant glioma. Oncol. Lett..

[41] Liu P.F., Chen C.F., Shu C.W., Chang H.M., Lee C.H., Liou H.H., Ger L.P., Chen C.L., Kang B.H. (2020). UBE2C is a Potential Biomarker for Tumorigenesis and Prognosis in Tongue Squamous Cell Carcinoma. Diagnostics.

[42] Zhang Z., Liu P., Wang J., Gong T., Zhang F., Ma J., Han N. (2015). Ubiquitin-conjugating enzyme E2C regulates apoptosis-dependent tumor progression of non-small cell lung cancer via ERK pathway. Med. Oncol..

[43] Li J., Zhi X., Shen X., Chen C., Yuan L., Dong X., Zhu C., Yao L., Chen M. (2020). Depletion of UBE2C reduces ovarian cancer malignancy and reverses cisplatin resistance via downregulating CDK1. Biochem. Biophys. Res. Commun..

[44] Lee J.Y., Tokumoto M., Fujiwara Y., Hasegawa T., Seko Y., Shimada A., Satoh M. (2016). Accumulation of p53 via down-regulation of *UBE2D* family genes is a critical pathway for cadmium-induced renal toxicity. Sci. Rep..

[45] Guan G.G., Wang W.B., Lei B.X., Wang Q.L., Wu L., Fu Z.M., Zhou F.X., Zhou Y.F. (2015). UBE2D3 is a positive prognostic factor and is negatively correlated with hTERT expression in esophageal cancer. Oncol. Lett..

[46] Luo H., Qin Y., Reu F., Ye S., Dai Y., Huang J., Wang F., Zhang D., Pan L., Zhu H. (2016). Microarray-based analysis and clinical validation identify ubiquitin-conjugating enzyme E2E1 (UBE2E1) as a prognostic factor in acute myeloid leukemia. J. Hematol. Oncol..

[47] Desai S.D., Reed R.E., Burks J., Wood L.M., Pullikuth A.K., Haas A.L., Liu L.F., Breslin J.W., Meiners S., Sankar S. (2012). ISG15 disrupts cytoskeletal architecture and promotes motility in human breast cancer cells. Exp. Biol. Med..

[48] Plafker K.S., Zyla K., Berry W., Plafker S.M. (2018). Loss of the ubiquitin conjugating enzyme UBE2E3 induces cellular senescence. Redox Biol..

[49] Debonneville C., Staub O. (2004). Participation of the ubiquitin-conjugating enzyme UBE2E3 in Nedd4-2-dependent regulation of the epithelial Na+ channel. Mol. Cell Biol..

[50] Liu R., Cheng Q., Song X., Wang H., Wang X., Wang L., Zhu B., Song L. (2019). A vital ubiquitin-conjugating enzyme CgUbe2g1 participated in regulation of immune response of Pacific oyster Crassostrea gigas. Dev. Comp. Immunol..

[51] Lu G., Weng S., Matyskiela M., Zheng X., Fang W., Wood S., Surka C., Mizukoshi R., Lu C.C., Mendy D. (2018). UBE2G1 governs the destruction of cereblon neomorphic substrates. eLife.

[52] Van de Weijer M.L., Schuren A.B.C., van den Boomen D.J.H., Mulder A., Claas F.H.J., Lehner P.J., Lebbink R.J., Wiertz E. (2017). Multiple E2 ubiquitin-conjugating enzymes regulate human cytomegalovirus US2-mediated immunoreceptor downregulation. J. Cell Sci..

[53] Zhao X., Yongchun Z., Qian H., Sanhui G., Jie L., Hong Y., Yanfei Z., Guizhen W., Yunchao H., Guangbiao Z. (2020). Identification of a potential tumor suppressor gene, *UBL3*, in non-small cell lung cancer. Cancer Biol. Med..

[54] Chen Z., Hu H. (2019). Identification of prognosis biomarkers of prostatic cancer in a cohort of 498 patients from TCGA. Curr. Probl. Cancer.

[55] Vourc’h P., Martin I., Bonnet-Brilhault F., Marouillat S., Barthélémy C., Pierre Müh J., Andres C. (2003). Mutation screening and association study of the UBE2H gene on chromosome 7q32 in autistic disorder. Psychiatry Genet..

[56] Li Y.P., Lecker S.H., Chen Y., Waddell I.D., Goldberg A.L., Reid M.B. (2003). TNF-alpha increases ubiquitin-conjugating activity in skeletal muscle by up-regulating UbcH2/E220k. FASEB J..

[57] Feng T., Deng L., Lu X., Pan W., Wu Q., Dai J. (2018). Ubiquitin-conjugating enzyme UBE2J1 negatively modulates interferon pathway and promotes RNA virus infection. Virol. J..

[58] Palmer C.J., Galan-Caridad J.M., Weisberg S.P., Lei L., Esquilin J.M., Croft G.F., Wainwright B., Canoll P., Owens D.M., Reizis B. (2014). Zfx facilitates tumorigenesis caused by activation of the Hedgehog pathway. Cancer Res..

[59] Chen S., Tan Y., Deng H., Shen Z., Liu Y., Wu P., Tan C., Jiang Y. (2017). UBE2J2 promotes hepatocellular carcinoma cell epithelial-mesenchymal transition and invasion in vitro. Oncotarget.

[60] Tao N.N., Zhang Z.Z., Ren J.H., Zhang J., Zhou Y.J., Wai Wong V.K., Kwan Law B.Y., Cheng S.T., Zhou H.Z., Chen W.X. (2020). Overexpression of ubiquitin-conjugating enzyme E2 L3 in hepatocellular carcinoma potentiates apoptosis evasion by inhibiting the GSK3β/p65 pathway. Cancer Lett..

[61] Yi S.A., Kim G.W., Yoo J., Han J.W., Kwon S.H. (2020). HP1γ Sensitizes Cervical Cancer Cells to Cisplatin through the Suppression of UBE2L3. Int. J. Mol. Sci..

[62] Ma X., Zhao J., Yang F., Liu H., Qi W. (2017). Ubiquitin conjugating enzyme E2 L3 promoted tumor growth of NSCLC through accelerating p27kip1 ubiquitination and degradation. Oncotarget.

[63] Whitcomb E.A., Tsai Y.C., Basappa J., Liu K., Le Feuvre A.K., Weissman A.M., Taylor A. (2019). Stabilization of p27(Kip1)/CDKN1B by UBCH7/UBE2L3 catalyzed ubiquitinylation: A new paradigm in cell-cycle control. FASEB J..

[64] Fu B., Li S., Wang L., Berman M.A., Dorf M.E. (2014). The ubiquitin conjugating enzyme UBE2L3 regulates TNFα-induced linear ubiquitination. Cell Res..

[65] Alpi A.F., Chaugule V., Walden H. (2016). Mechanism and disease association of E2-conjugating enzymes: Lessons from UBE2T and UBE2L3. Biochem. J..

[66] Hodge C.D., Edwards R.A., Markin C.J., McDonald D., Pulvino M., Huen M.S., Zhao J., Spyracopoulos L., Hendzel M.J., Glover J.N. (2015). Covalent Inhibition of Ubc13 Affects Ubiquitin Signaling and Reveals Active Site Elements Important for Targeting. ACS Chem. Biol..

[67] Song T.T., Xu F., Wang W. (2020). Inhibiting ubiquitin conjugating enzyme *E2 N* by microRNA-590-3p reduced cell growth of cervical carcinoma. Kaohsiung J. Med. Sci..

[68] Zhang E., Liu Q., Wang Y., Wang H., He L., Jin X., Li N. (2017). MicroRNA miR-147b promotes tumor growth via targeting *UBE2N* in hepatocellular carcinoma. Oncotarget.

[69] Dikshit A., Jin Y.J., Degan S., Hwang J., Foster M.W., Li C.Y., Zhang J.Y. (2018). UBE2N Promotes Melanoma Growth via MEK/FRA1/SOX10 Signaling. Cancer Res..

[70] Shen T., Cai L.D., Liu Y.H., Li S., Gan W.J., Li X.M., Wang J.R., Guo P.D., Zhou Q., Lu X.X. (2018). Ube2v1-mediated ubiquitination and degradation of Sirt1 promotes metastasis of colorectal cancer by epigenetically suppressing autophagy. J. Hematol. Oncol..

[71] Chen S., Yang J., Zhang Y., Duan C., Liu Q., Huang Z., Xu Y., Zhou L., Xu G. (2018). Ubiquitin-conjugating enzyme UBE2O regulates cellular clock function by promoting the degradation of the transcription factor BMAL1. J. Biol. Chem..

[72] Liu X., Ma F., Liu C., Zhu K., Li W., Xu Y., Li G., Niu Z., Liu J., Chen D. (2020). UBE2O promotes the proliferation, EMT and stemness properties of breast cancer cells through the UBE2O/AMPKα2/mTORC1-MYC positive feedback loop. Cell Death Dis..

[73] Chen X., Zhang S., Liu C., Li G., Lu S., Wang Y., Zhang X., Huang D., Qiu Y., Liu Y. (2020). UBE2O Promotes Progression and Epithelial-Mesenchymal Transition in Head and Neck Squamous Cell Carcinoma. OncoTargets Ther..

[74] Huang Y., Yang X., Lu Y., Zhao Y., Meng R., Zhang S., Dong X., Xu S., Wu G. (2021). UBE2O targets Mxi1 for ubiquitination and degradation to promote lung cancer progression and radioresistance. Cell Death Differ..

[75] Nguyen A.T., Prado M.A., Schmidt P.J., Sendamarai A.K., Wilson-Grady J.T., Min M., Campagna D.R., Tian G., Shi Y., Dederer V. (2017). UBE2O remodels the proteome during terminal erythroid differentiation. Science.

[76] Zhang X., Zhang J., Bauer A., Zhang L., Selinger D.W., Lu C.X., Ten Dijke P. (2013). Fine-tuning BMP7 signalling in adipogenesis by UBE2O/E2-230K-mediated monoubiquitination of SMAD6. EMBO J..

[77] Shafiee S.M., Rasti M., Seghatoleslam A., Azimi T., Owji A.A. (2015). UBE2Q1 in a Human Breast Carcinoma Cell Line: Overexpression and Interaction with p53. Asian Pac. J. Cancer Prev..

[78] Seghatoleslam A., Bozorg-Ghalati F., Monabati A., Nikseresht M., Owji A.A. (2014). *UBE2Q1*, as a Down Regulated Gene in Pediatric Acute Lymphoblastic Leukemia. Int. J. Mol. Cell Med..

[79] Shafiee S.M., Seghatoleslam A., Nikseresht M., Hosseini S.V., Alizadeh-Naeeni M., Safaei A., Owji A.A. (2013). UBE2Q1 expression in human colorectal tumors and cell lines. Mol. Biol. Rep..

[80] Kravic B., Behrends C., Meyer H. (2020). Regulation of lysosome integrity and lysophagy by the ubiquitin-conjugating enzyme UBE2QL1. Autophagy.

[81] Maeda H., Miyajima N., Kano S., Tsukiyama T., Okumura F., Fukuda S., Hatakeyama S. (2009). Ubiquitin-conjugating enzyme UBE2Q2 suppresses cell proliferation and is down-regulated in recurrent head and neck cancer. Mol. Cancer Res..

[82] Shafiee S.M., Seghatoleslam A., Nikseresht M., Hosseini S.V., Alizadeh-Naeeni M., Safaei A., Owji A.A. (2014). Expression Status of UBE2Q2 in Colorectal Primary Tumors and Cell Lines. Iran. J. Med. Sci..

[83] Williams K.M., Qie S., Atkison J.H., Salazar-Arango S., Alan Diehl J., Olsen S.K. (2019). Structural insights into E1 recognition and the ubiquitin-conjugating activity of the E2 enzyme Cdc34. Nat. Commun..

[84] Liu X., Zhang Y., Hu Z., Li Q., Yang L., Xu G. (2018). The Catalytically Inactive Mutation of the Ubiquitin-Conjugating Enzyme CDC34 Affects its Stability and Cell Proliferation. Protein J..

[85] Zhao X.C., Wang G.Z., Wen Z.S., Zhou Y.C., Hu Q., Zhang B., Qu L.W., Gao S.H., Liu J., Ma L. (2020). Systematic identification of CDC34 that functions to stabilize EGFR and promote lung carcinogenesis. eBioMedicine.

[86] Eliseeva E., Pati D., Diccinanni M.B., Yu A.L., Mohsin S.K., Margolin J.F., Plon S.E. (2001). Expression and localization of the CDC34 ubiquitin-conjugating enzyme in pediatric acute lymphoblastic leukemia. Cell Growth Differ..

[87] Yoshimura S., Kasamatsu A., Nakashima D., Iyoda M., Kasama H., Saito T., Takahara T., Endo-Sakamoto Y., Shiiba M., Tanzawa H. (2017). UBE2S associated with OSCC proliferation by promotion of P21 degradation via the ubiquitin-proteasome system. Biochem. Biophys. Res. Commun..

[88] Ayesha A.K., Hyodo T., Asano E., Sato N., Mansour M.A., Ito S., Hamaguchi M., Senga T. (2016). UBE2S is associated with malignant characteristics of breast cancer cells. Tumour Biol..

[89] Wang J., Zhang Y., Hou J., Qian X., Zhang H., Zhang Z., Li M., Wang R., Liao K., Wang Y. (2016). Ube2s regulates Sox2 stability and mouse ES cell maintenance. Cell Death Differ..

[90] Kelsall I.R., Langenick J., MacKay C., Patel K.J., Alpi A.F. (2012). The Fanconi anaemia components UBE2T and FANCM are functionally linked to nucleotide excision repair. PLoS ONE.

[91] Hira A., Yoshida K., Sato K., Okuno Y., Shiraishi Y., Chiba K., Tanaka H., Miyano S., Shimamoto A., Tahara H. (2015). Mutations in the gene encoding the E2 conjugating enzyme UBE2T cause Fanconi anemia. Am. J. Hum. Genet..

[92] Yu H., Xiang P., Pan Q., Huang Y., Xie N., Zhu W. (2016). Ubiquitin-Conjugating Enzyme E2T is an Independent Prognostic Factor and Promotes Gastric Cancer Progression. Tumour Biol..

[93] Wang Y., Leng H., Chen H., Wang L., Jiang N., Huo X., Yu B. (2016). Knockdown of UBE2T Inhibits Osteosarcoma Cell Proliferation, Migration, and Invasion by Suppressing the PI3K/Akt Signaling Pathway. Oncol Res..

[94] Wen M., Kwon Y., Wang Y., Mao J.H., Wei G. (2015). Elevated expression of UBE2T exhibits oncogenic properties in human prostate cancer. Oncotarget.

[95] Wu Z.H., Zhang Y.J., Sun H.Y. (2020). High ubiquitin conjugating enzyme *E2 T* mRNA expression and its prognostic significance in lung adenocarcinoma: A study based on the TCGA database. Medicine.

[96] Perez-Peña J., Corrales-Sánchez V., Amir E., Pandiella A., Ocana A. (2017). Ubiquitin-conjugating enzyme E2T (UBE2T) and denticleless protein homolog (DTL) are linked to poor outcome in breast and lung cancers. Sci. Rep..

[97] Liu L.L., Zhu J.M., Yu X.N., Zhu H.R., Shi X., Bilegsaikhan E., Guo H.Y., Wu J., Shen X.Z. (2019). UBE2T promotes proliferation via G2/M checkpoint in hepatocellular carcinoma. Cancer Manag. Res..

[98] Yu H., Wang H., Dong W., Cao Z.Y., Li R., Yang C., Cong W.M., Dong H., Jin G.Z. (2020). The diagnostic and prognostic value of UBE2T in intrahepatic cholangiocarcinoma. PeerJ.

[99] Zhu X., Li T., Niu X., Chen L., Ge C. (2020). Identification of UBE2T as an independent prognostic biomarker for gallbladder cancer. Oncol. Lett..

[100] Zhang W., Zhang Y., Yang Z., Liu X., Yang P., Wang J., Hu K., He X., Zhang X., Jing H. (2019). High expression of UBE2T predicts poor prognosis and survival in multiple myeloma. Cancer Gene Ther..

[101] Zou R., Xu H., Li F., Wang S., Zhu L. (2021). Increased Expression of UBE2T Predicting Poor Survival of Epithelial Ovarian Cancer: Based on Comprehensive Analysis of UBE2s, Clinical Samples, and the GEO Database. DNA Cell Biol..

[102] Liu L.P., Yang M., Peng Q.Z., Li M.Y., Zhang Y.S., Guo Y.H., Chen Y., Bao S.Y. (2017). UBE2T promotes hepatocellular carcinoma cell growth via ubiquitination of p53. Biochem. Biophys. Res. Commun..

[103] Zhang W., Zhuang Y., Zhang Y., Yang X., Zhang H., Wang G., Yin W., Wang R., Zhang Z., Xiao W. (2017). Uev1A facilitates osteosarcoma differentiation by promoting Smurf1-mediated Smad1 ubiquitination and degradation. Cell Death Dis..

[104] Lei B., Xie L., Zhang S., Lv D., Shu F., Deng Y. (2020). UBE2W down-regulation promotes cell apoptosis and correlates with hypospermatogenesis. Andrologia.

[105] Ikeda F. (2018). The anti-apoptotic ubiquitin conjugating enzyme BIRC6/BRUCE regulates autophagosome-lysosome fusion. Autophagy.

[106] Tang W., Xue R., Weng S., Wu J., Fang Y., Wang Y., Ji L., Hu T., Liu T., Huang X. (2015). BIRC6 promotes hepatocellular carcinogenesis: Interaction of BIRC6 with p53 facilitating p53 degradation. Int. J. Cancer.

[107] Yang G., Wang X., Liu B., Lu Z., Xu Z., Xiu P., Liu Z., Li J. (2019). *circ-BIRC6*, a circular RNA, promotes hepatocellular carcinoma progression by targeting the miR-3918/Bcl2 axis. Cell Cycle.

[108] Lamers F., Schild L., Koster J., Speleman F., Øra I., Westerhout E.M., van Sluis P., Versteeg R., Caron H.N., Molenaar J.J. (2012). Identification of BIRC6 as a novel intervention target for neuroblastoma therapy. BMC Cancer.

[109] Zhuang W., Zhang C., Hao F., Sun X. (2018). Baculoviral IAP Repeat Containing 6 (BIRC6) Is a Predictor of Prognosis in Prostate Cancer. Med. Sci. Monit..

[110] Zhou W., Xu J., Li H., Xu M., Chen Z.J., Wei W., Pan Z., Sun Y. (2017). Neddylation E2 UBE2F Promotes the Survival of Lung Cancer Cells by Activating CRL5 to Degrade NOXA via the K11 Linkage. Clin. Cancer Res..

[111] Wang A., Ding X., Demarque M., Liu X., Pan D., Xin H., Zhong B., Wang X., Dejean A., Jin W. (2017). Ubc9 Is Required for Positive Selection and Late-Stage Maturation of Thymocytes. J. Immunol..

[112] Wang F., Sun F., Luo J., Yue T., Chen L., Zhou H., Zhang J., Yang C., Luo X., Zhou Q. (2019). Loss of ubiquitin-conjugating enzyme E2 (Ubc9) in macrophages exacerbates multiple low-dose streptozotocin-induced diabetes by attenuating M2 macrophage polarization. Cell Death Dis..

[113] Varadaraj A., Mattoscio D., Chiocca S. (2014). SUMO Ubc9 enzyme as a viral target. IUBMB Life.

[114] Fang S., Qiu J., Wu Z., Bai T., Guo W. (2017). Down-regulation of UBC9 increases the sensitivity of hepatocellular carcinoma to doxorubicin. Oncotarget.

[115] Zong Y., Wu P., Nai C., Luo Y., Hu F., Gao W., Zhai N., Xu T., Li D. (2017). Effect of MicroRNA-30e on the Behavior of Vascular Smooth Muscle Cells via Targeting Ubiquitin-Conjugating Enzyme *E2I*. Circ. J..

[116] McManus F.P., Bourdeau V., Acevedo M., Lopes-Paciencia S., Mignacca L., Lamoliatte F., Rojas Pino J.W., Ferbeyre G., Thibault P. (2018). Quantitative SUMO proteomics reveals the modulation of several PML nuclear body associated proteins and an anti-senescence function of UBC9. Sci. Rep..

[117] Lu Z., Wu H., Mo Y.Y. (2006). Regulation of bcl-2 expression by Ubc9. Exp. Cell Res..

[118] Jo S., Lee Y.L., Kim S., Lee H., Chung H. (2016). PCGF2 negatively regulates arsenic trioxide-induced PML-RARA protein degradation via UBE2I inhibition in NB4 cells. Biochim. Biophys. Acta.

[119] Edrees M.A.H., Luo J., Sun F., Wang F., He L., Yue T., Chen L., Zhang J., Zhou H., Yang C. (2019). *Ubc9* deficiency selectively impairs the functionality of common lymphoid progenitors (CLPs) during bone marrow hematopoiesis. Mol. Immunol..

[120] Cukras S., Morffy N., Ohn T., Kee Y. (2014). Inactivating UBE2M impacts the DNA damage response and genome integrity involving multiple cullin ligases. PLoS ONE.

[121] Zhang G.C., Yu X.N., Sun J.L., Xiong J., Yang Y.J., Jiang X.M., Zhu J.M. (2020). UBE2M promotes cell proliferation via the β-catenin/cyclin D1 signaling in hepatocellular carcinoma. Aging.

[122] Xu B., Deng Y., Bi R., Guo H., Shu C., Shah N.K., Chang J., Liu G., Du Y., Wei W. (2018). A first-in-class inhibitor, MLN4924 (pevonedistat), induces cell-cycle arrest, senescence, and apoptosis in human renal cell carcinoma by suppressing UBE2M-dependent neddylation modification. Cancer Chemother. Pharmacol..

[123] Zhao B., Gao C., Shi D., Mao J., Zhao J., Guo L., Guo J., Jiao Z. (2019). Knockdown of Nedd8-conjugating enzyme *UBE2M* suppresses the proliferation and induces the apoptosis of intrahepatic cholangiocarcinoma cells. Oncol. Rep..

[124] Zhou W., Xu J., Tan M., Li H., Li H., Wei W., Sun Y. (2018). UBE2M Is a Stress-Inducible Dual E2 for Neddylation and Ubiquitylation that Promotes Targeted Degradation of UBE2F. Mol. Cell.

[125] Li L., Kang J., Zhang W., Cai L., Wang S., Liang Y., Jiang Y., Liu X., Zhang Y., Ruan H. (2019). Validation of NEDD8-conjugating enzyme UBC12 as a new therapeutic target in lung cancer. eBioMedicine.

[126] Shi X., Wang B., Chen X., Zheng Y., Ding Y., Wang C. (2020). Upregulation of ubiquitin-conjugating enzyme E2Z is associated with human hepatocellular carcinoma. Biochem. Biophys. Res. Commun..

[127] Hofmann K. (2009). Ubiquitin-binding domains and their role in the DNA damage response. DNA Repair.

[128] Akita M., Tak Y.S., Shimura T., Matsumoto S., Okuda-Shimizu Y., Shimizu Y., Nishi R., Saitoh H., Iwai S., Mori T. (2015). SUMOylation of xeroderma pigmentosum group C protein regulates DNA damage recognition during nucleotide excision repair. Sci. Rep..

[129] Sun Y., Miller Jenkins L.M., Su Y.P., Nitiss K.C., Nitiss J.L., Pommier Y. (2020). A conserved SUMO pathway repairs topoisomerase DNA-protein cross-links by engaging ubiquitin-mediated proteasomal degradation. Sci. Adv..

[130] Kaiser P., Mansour H.A., Greeten T., Auer B., Schweiger M., Schneider R. (1994). The human ubiquitin-conjugating enzyme UbcH1 is involved in the repair of UV-damaged, alkylated and cross-linked DNA. FEBS Lett..

[131] Roos W.P., Thomas A.D., Kaina B. (2016). DNA damage and the balance between survival and death in cancer biology. Nat. Rev. Cancer.

[132] Torres-Ramos C.A., Prakash S., Prakash L. (2002). Requirement of RAD5 and MMS2 for postreplication repair of UV-damaged DNA in Saccharomyces cerevisiae. Mol. Cell Biol..

[133] Hoege C., Pfander B., Moldovan G.L., Pyrowolakis G., Jentsch S. (2002). RAD6-dependent DNA repair is linked to modification of PCNA by ubiquitin and SUMO. Nature.

[134] Lentucci C., Belkina A.C., Cederquist C.T., Chan M., Johnson H.E., Prasad S., Lopacinski A., Nikolajczyk B.S., Monti S., Snyder-Cappione J. (2017). Inhibition of Ubc13-mediated Ubiquitination by GPS2 Regulates Multiple Stages of B Cell Development. J. Biol. Chem..

[135] Burma S., Chen B.P., Chen D.J. (2006). Role of non-homologous end joining (NHEJ) in maintaining genomic integrity. DNA Repair.

[136] Hu L., Li X., Liu Q., Xu J., Ge H., Wang Z., Wang H., Wang Z., Shi C., Xu X. (2017). UBE2S, a novel substrate of Akt1, associates with Ku70 and regulates DNA repair and glioblastoma multiforme resistance to chemotherapy. Oncogene.

[137] An H., Yang L., Wang C., Gan Z., Gu H., Zhang T., Huang X., Liu Y., Li Y., Chang S.J. (2017). Interactome Analysis Reveals a Novel Role for RAD6 in the Regulation of Proteasome Activity and Localization in Response to DNA Damage. Mol. Cell Biol..

[138] Machida Y.J., Machida Y., Chen Y., Gurtan A.M., Kupfer G.M., D’Andrea A.D., Dutta A. (2006). UBE2T is the E2 in the Fanconi anemia pathway and undergoes negative autoregulation. Mol. Cell.

[139] Nijman S.M., Huang T.T., Dirac A.M., Brummelkamp T.R., Kerkhoven R.M., D’Andrea A.D., Bernards R. (2005). The deubiquitinating enzyme USP1 regulates the Fanconi anemia pathway. Mol. Cell.

[140] Mamrak N.E., Shimamura A., Howlett N.G. (2017). Recent discoveries in the molecular pathogenesis of the inherited bone marrow failure syndrome Fanconi anemia. Blood Rev..

[141] Rickman K.A., Lach F.P., Abhyankar A., Donovan F.X., Sanborn E.M., Kennedy J.A., Sougnez C., Gabriel S.B., Elemento O., Chandrasekharappa S.C. (2015). Deficiency of *UBE2T*, the E2 Ubiquitin Ligase Necessary for FANCD2 and FANCI Ubiquitination, Causes FA-T Subtype of Fanconi Anemia. Cell Rep..

[142] Lyakhovich A., Surralles J. (2007). *FANCD2* depletion sensitizes cancer cells repopulation ability in vitro. Cancer Lett..

[143] Ramaekers C.H., van den Beucken T., Meng A., Kassam S., Thoms J., Bristow R.G., Wouters B.G. (2011). Hypoxia disrupts the Fanconi anemia pathway and sensitizes cells to chemotherapy through regulation of UBE2T. Radiother. Oncol..

[144] Alagpulinsa D.A., Kumar S., Talluri S., Nanjappa P., Buon L., Chakraborty C., Samur M.K., Szalat R., Shammas M.A., Munshi N.C. (2019). Amplification and overexpression of E2 ubiquitin conjugase UBE2T promotes homologous recombination in multiple myeloma. Blood Adv..

[145] Tarsounas M., Sung P. (2020). The antitumorigenic roles of BRCA1-BARD1 in DNA repair and replication. Nat. Rev. Mol. Cell Biol..

[146] Kothayer H., Spencer S.M., Tripathi K., Westwell A.D., Palle K. (2016). Synthesis and in vitro anticancer evaluation of some 4,6-diamino-1,3,5-triazine-2-carbohydrazides as Rad6 ubiquitin conjugating enzyme inhibitors. Bioorg. Med. Chem. Lett..

[147] Somasagara R.R., Spencer S.M., Tripathi K., Clark D.W., Mani C., Madeira da Silva L., Scalici J., Kothayer H., Westwell A.D., Rocconi R.P. (2017). RAD6 promotes DNA repair and stem cell signaling in ovarian cancer and is a promising therapeutic target to prevent and treat acquired chemoresistance. Oncogene.

[148] Clark D.W., Mani C., Palle K. (2018). RAD6 promotes chemoresistance in ovarian cancer. Mol. Cell. Oncol..

[149] Hsu S.H., Chen S.H., Kuo C.C., Chang J.Y. (2018). Ubiquitin-conjugating enzyme E2 B regulates the ubiquitination of O(6)-methylguanine-DNA methyltransferase and BCNU sensitivity in human nasopharyngeal carcinoma cells. Biochem. Pharmacol..

[150] Yang H., Wu L., Ke S., Wang W., Yang L., Gao X., Fang H., Yu H., Zhong Y., Xie C. (2016). Downregulation of Ubiquitin-conjugating Enzyme UBE2D3 Promotes Telomere Maintenance and Radioresistance of Eca-109 Human Esophageal Carcinoma Cells. J. Cancer.

[151] Zhou Y., Chen R., Luo X., Zhang W.D., Qin J.J. (2020). The E2 ubiquitin-conjugating enzyme UbcH5c: An emerging target in cancer and immune disorders. Drug Discov. Today.

[152] Lydeard J.R., Schulman B.A., Harper J.W. (2013). Building and remodelling Cullin-RING E3 ubiquitin ligases. EMBO Rep..

[153] Yoon H., Kim M., Jang K., Shin M., Besser A., Xiao X., Zhao D., Wander S.A., Briegel K., Morey L. (2019). p27 transcriptionally coregulates cJun to drive programs of tumor progression. Proc. Natl. Acad. Sci. USA.

[154] Mittal M.K., Singh K., Misra S., Chaudhuri G. (2011). SLUG-induced elevation of D1 cyclin in breast cancer cells through the inhibition of its ubiquitination. J. Biol. Chem..

[155] Pierce N.W., Lee J.E., Liu X., Sweredoski M.J., Graham R.L., Larimore E.A., Rome M., Zheng N., Clurman B.E., Hess S. (2013). Cand1 promotes assembly of new SCF complexes through dynamic exchange of F box proteins. Cell.

[156] Walker A., Acquaviva C., Matsusaka T., Koop L., Pines J. (2008). UbcH10 has a rate-limiting role in G1 phase but might not act in the spindle checkpoint or as part of an autonomous oscillator. J. Cell Sci..

[157] Cai F., Chen P., Chen L., Biskup E., Liu Y., Chen P.C., Chang J.F., Jiang W., Jing Y., Chen Y. (2014). Human RAD6 promotes G1-S transition and cell proliferation through upregulation of cyclin D1 expression. PLoS ONE.

[158] Eifler K., Vertegaal A.C.O. (2015). SUMOylation-Mediated Regulation of Cell Cycle Progression and Cancer. Trends Biochem. Sci..

[159] Bellail A.C., Olson J.J., Hao C. (2014). SUMO1 modification stabilizes CDK6 protein and drives the cell cycle and glioblastoma progression. Nat. Commun..

[160] Block K., Boyer T.G., Yew P.R. (2001). Phosphorylation of the human ubiquitin-conjugating enzyme, CDC34, by casein kinase. J. Biol. Chem..

[161] Ciliberto A., Shah J.V. (2009). A quantitative systems view of the spindle assembly checkpoint. EMBO J..

[162] Wild T., Larsen M.S., Narita T., Schou J., Nilsson J., Choudhary C. (2016). The Spindle Assembly Checkpoint Is Not Essential for Viability of Human Cells with Genetically Lowered APC/C Activity. Cell Rep..

[163] Reddy S.K., Rape M., Margansky W.A., Kirschner M.W. (2007). Ubiquitination by the anaphase-promoting complex drives spindle checkpoint inactivation. Nature.

[164] Ben-Eliezer I., Pomerantz Y., Galiani D., Nevo N., Dekel N. (2015). Appropriate expression of Ube2C and Ube2S controls the progression of the first meiotic division. FASEB J..

[165] Hao Z., Zhang H., Cowell J. (2012). Ubiquitin-conjugating enzyme UBE2C: Molecular biology, role in tumorigenesis, and potential as a biomarker. Tumour. Biol..

[166] Voutsadakis I.A. (2013). Ubiquitin- and ubiquitin-like proteins-conjugating enzymes (E2s) in breast cancer. Mol. Biol. Rep..

[167] Bremm A., Komander D. (2011). Emerging roles for Lys11-linked polyubiquitin in cellular regulation. Trends. Biochem. Sci..

[168] Saville M.K., Sparks A., Xirodimas D.P., Wardrop J., Stevenson L.F., Bourdon J.C., Woods Y.L., Lane D.P. (2004). Regulation of p53 by the ubiquitin-conjugating enzymes UbcH5B/C in vivo. J. Biol. Chem..

[169] Wu M., Li X., Huang W., Chen Y., Wang B., Liu X. (2020). Ubiquitin-conjugating enzyme E2T(UBE2T) promotes colorectal cancer progression by facilitating ubiquitination and degradation of p53. Clin. Res. Hepatol. Gastroenterol..

[170] Taylor W.R., Stark G.R. (2001). Regulation of the G2/M transition by p53. Oncogene.

[171] Fokas E., O’Neill E., Gordon-Weeks A., Mukherjee S., McKenna W.G., Muschel R.J. (2015). Pancreatic ductal adenocarcinoma: From genetics to biology to radiobiology to oncoimmunology and all the way back to the clinic. Biochim. Biophys. Acta.

[172] Pan Y.H., Yang M., Liu L.P., Wu D.C., Li M.Y., Su S.G. (2018). UBE2S enhances the ubiquitination of p53 and exerts oncogenic activities in hepatocellular carcinoma. Biochem. Biophys. Res. Commun..

[173] Hong N.H., Tak Y.J., Rhim H., Kang S. (2019). Hip2 ubiquitin-conjugating enzyme has a role in UV-induced G1/S arrest and re-entry. Genes Genom..

[174] Bae Y., Jung S.H., Kim G.Y., Rhim H., Kang S. (2013). Hip2 ubiquitin-conjugating enzyme overcomes radiation-induced G2/M arrest. Biochim. Biophys. Acta.

[175] Zhang B., Deng C., Wang L., Zhou F., Zhang S., Kang W., Zhan P., Chen J., Shen S., Guo H. (2018). Upregulation of UBE2Q1 via gene copy number gain in hepatocellular carcinoma promotes cancer progression through β-catenin-EGFR-PI3K-Akt-mTOR signaling pathway. Mol. Carcinog..

[176] Wang P., Li Y., Ma Y., Zhang X., Li Z., Yu W., Zhu M., Wang J., Xu Y., Xu A. (2021). Comprehensive Investigation into the Role of Ubiquitin-Conjugating Enzyme E2S in Melanoma Development. J. Invest. Dermatol..

[177] Gong Y.Q., Peng D., Ning X.H., Yang X.Y., Li X.S., Zhou L.Q., Guo Y.L. (2016). *UBE2T* silencing suppresses proliferation and induces cell cycle arrest and apoptosis in bladder cancer cells. Oncol. Lett..

[178] Luo C., Yao Y., Yu Z., Zhou H., Guo L., Zhang J., Cao H., Zhang G., Li Y., Jiao Z. (2017). *UBE2T* knockdown inhibits gastric cancer progression. Oncotarget.

[179] Palumbo A., Da Costa N.M., De Martino M., Sepe R., Pellecchia S., de Sousa V.P., Nicolau Neto P., Kruel C.D., Bergman A., Nasciutti L.E. (2016). UBE2C is overexpressed in ESCC tissues and its abrogation attenuates the malignant phenotype of ESCC cell lines. Oncotarget.

[180] Nicolau-Neto P., Palumbo A., De Martino M., Esposito F., de Almeida Simão T., Fusco A., Nasciutti L.E., Meireles Da Costa N., Ribeiro Pinto L.F. (2018). *UBE2C* Is a Transcriptional Target of the Cell Cycle Regulator FOXM. Genes.

[181] Liu Y., Zhao R., Chi S., Zhang W., Xiao C., Zhou X., Zhao Y., Wang H. (2020). UBE2C Is Upregulated by Estrogen and Promotes Epithelial-Mesenchymal Transition via p53 in Endometrial Cancer. Mol. Cancer Res..

[182] Wang X., Yin L., Yang L., Zheng Y., Liu S., Yang J., Cui H., Wang H. (2019). Silencing *ubiquitin-conjugating enzyme 2C* inhibits proliferation and epithelial-mesenchymal transition in pancreatic ductal adenocarcinoma. FEBS J..

[183] Huang P., Guo Y., Zhao Z., Ning W., Wang H., Gu C., Zhang M., Qu Y., Zhang H., Song Y. (2020). UBE2T promotes glioblastoma invasion and migration via stabilizing GRP78 and regulating EMT. Aging.

[184] Bisol Â., de Campos P.S., Lamers M.L. (2020). Flavonoids as anticancer therapies: A systematic review of clinical trials. Phytother. Res..

[185] Van Cruchten S., Van Den Broeck W. (2002). Morphological and biochemical aspects of apoptosis, oncosis and necrosis. Anat. Histol. Embryol..

[186] Jesenberger V., Jentsch S. (2002). Deadly encounter: Ubiquitin meets apoptosis. Nat. Rev. Mol. Cell Biol..

[187] Aharinejad S., Andrukhova O., Lucas T., Zuckermann A., Wieselthaler G., Wolner E., Grimm M. (2008). Programmed cell death in idiopathic dilated cardiomyopathy is mediated by suppression of the apoptosis inhibitor Apollon. Ann. Thorac. Surg..

[188] Bartke T., Pohl C., Pyrowolakis G., Jentsch S. (2004). Dual role of BRUCE as an antiapoptotic IAP and a chimeric E2/E3 ubiquitin ligase. Mol. Cell.

[189] Hu T., Weng S., Tang W., Xue R., Chen S., Cai G., Cai Y., Shen X., Zhang S., Dong L. (2015). Overexpression of BIRC6 Is a Predictor of Prognosis for Colorectal Cancer. PLoS ONE.

[190] Low C.G., Luk I.S., Lin D., Fazli L., Yang K., Xu Y., Gleave M., Gout P.W., Wang Y. (2013). BIRC6 protein, an inhibitor of apoptosis: Role in survival of human prostate cancer cells. PLoS ONE.

[191] Dong X., Lin D., Low C., Vucic E.A., English J.C., Yee J., Murray N., Lam W.L., Ling V., Lam S. (2013). Elevated expression of BIRC6 protein in non-small-cell lung cancers is associated with cancer recurrence and chemoresistance. J. Thorac. Oncol..

[192] Ismail E.A., Mahmoud H.M., Tawfik L.M., Habashy D.M., Adly A.A., El-Sherif N.H., Abdelwahab M.A. (2012). *BIRC6/Apollon* gene expression in childhood acute leukemia: Impact on therapeutic response and prognosis. Eur. J. Haematol..

[193] Luk S.U., Xue H., Cheng H., Lin D., Gout P.W., Fazli L., Collins C.C., Gleave M.E., Wang Y. (2014). The *BIRC6* gene as a novel target for therapy of prostate cancer: Dual targeting of inhibitors of apoptosis. Oncotarget.

[194] Xu Y., Zhang Z., Li J., Tong J., Cao B., Taylor P., Tang X., Wu D., Moran M.F., Zeng Y. (2017). The ubiquitin-conjugating enzyme UBE2O modulates c-Maf stability and induces myeloma cell apoptosis. J. Hematol. Oncol..

[195] Ba C., Ni X., Yu J., Zou G., Zhu H. (2020). Ubiquitin conjugating enzyme E2 M promotes apoptosis in osteoarthritis chondrocytes via Wnt/β-catenin signaling. Biochem. Biophys. Res. Commun..

[196] Sun X.X., Challagundla K.B., Dai M.S. (2012). Positive regulation of p53 stability and activity by the deubiquitinating enzyme Otubain. EMBO J..

[197] Ren J., Shi M., Liu R., Yang Q.H., Johnson T., Skarnes W.C., Du C. (2005). The *Birc6 (Bruce)* gene regulates p53 and the mitochondrial pathway of apoptosis and is essential for mouse embryonic development. Proc. Natl. Acad. Sci. USA.

[198] Zhou C., Bi F., Yuan J., Yang F., Sun S. (2018). Gain of UBE2D1 facilitates hepatocellular carcinoma progression and is associated with DNA damage caused by continuous IL-6. J. Exp. Clin. Cancer Res..

[199] Clevers H., Nusse R. (2012). Wnt/β-catenin signaling and disease. Cell.

[200] Schaefer K.N., Peifer M. (2019). Wnt/Beta-Catenin Signaling Regulation and a Role for Biomolecular Condensates. Dev. Cell.

[201] Li Z., Wang Y., Li Y., Yin W., Mo L., Qian X., Zhang Y., Wang G., Bu F., Zhang Z. (2018). Ube2s stabilizes β-Catenin through K11-linked polyubiquitination to promote mesendoderm specification and colorectal cancer development. Cell Death Dis..

[202] Shekhar M.P., Gerard B., Pauley R.J., Williams B.O., Tait L. (2008). Rad6B is a positive regulator of beta-catenin stabilization. Cancer Res..

[203] Shekhar M.P., Tait L., Gerard B. (2006). Essential role of T-cell factor/beta-catenin in regulation of Rad6B: A potential mechanism for Rad6B overexpression in breast cancer cells. Mol. Cancer Res..

[204] Shin S., Im H.J., Kwon Y.J., Ye D.J., Baek H.S., Kim D., Choi H.K., Chun Y.J. (2017). Human steroid sulfatase induces Wnt/β-catenin signaling and epithelial-mesenchymal transition by upregulating Twist1 and HIF-1α in human prostate and cervical cancer cells. Oncotarget.

[205] Qin Y., Du J., Fan C. (2020). Ube2S regulates Wnt/β-catenin signaling and promotes the progression of non-small cell lung cancer. Int. J. Med. Sci..

[206] Nusse R., Clevers H. (2017). Wnt/β-Catenin Signaling, Disease, and Emerging Therapeutic Modalities. Cell.

[207] Jung C.R., Hwang K.S., Yoo J., Cho W.K., Kim J.M., Kim W.H., Im D.S. (2006). E2-EPF UCP targets pVHL for degradation and associates with tumor growth and metastasis. Nat. Med..

[208] Lin M., Lei T., Zheng J., Chen S., Du L., Xie H. (2019). UBE2S mediates tumor progression via SOX6/β-Catenin signaling in endometrial cancer. Int. J. Biochem. Cell Biol..

[209] Liu J., Liu X. (2017). *UBE2T* silencing inhibited non-small cell lung cancer cell proliferation and invasion by suppressing the wnt/β-catenin signaling pathway. Int. J. Clin. Exp. Pathol..

[210] Hu W., Xiao L., Cao C., Hua S., Wu D. (2016). UBE2T promotes nasopharyngeal carcinoma cell proliferation, invasion, and metastasis by activating the AKT/GSK3β/β-catenin pathway. Oncotarget.

[211] Deng L., Meng T., Chen L., Wei W., Wang P. (2020). The role of ubiquitination in tumorigenesis and targeted drug discovery. Signal. Transduct. Target. Ther..

[212] Mitchell S., Vargas J., Hoffmann A. (2016). Signaling via the NFκB system. Wiley Interdiscip. Rev. Syst. Biol. Med..

[213] Magnani M., Crinelli R., Bianchi M., Antonelli A. (2000). The ubiquitin-dependent proteolytic system and other potential targets for the modulation of nuclear factor-kB (NF-kB). Curr. Drug Targets.

[214] Wu X., Karin M. (2015). Emerging roles of Lys63-linked polyubiquitylation in immune responses. Immunol. Rev..

[215] Varfolomeev E., Goncharov T., Fedorova A.V., Dynek J.N., Zobel K., Deshayes K., Fairbrother W.J., Vucic D. (2008). c-IAP1 and c-IAP2 are critical mediators of tumor necrosis factor alpha (TNFalpha)-induced NF-kappaB activation. J. Biol. Chem..

[216] Zhou H., Wertz I., O’Rourke K., Ultsch M., Seshagiri S., Eby M., Xiao W., Dixit V.M. (2004). Bcl10 activates the NF-kappaB pathway through ubiquitination of NEMO. Nature.

[217] Deng L., Wang C., Spencer E., Yang L., Braun A., You J., Slaughter C., Pickart C., Chen Z.J. (2000). Activation of the IkappaB kinase complex by TRAF6 requires a dimeric ubiquitin-conjugating enzyme complex and a unique polyubiquitin chain. Cell.

[218] Shi C.S., Kehrl J.H. (2003). Tumor necrosis factor (TNF)-induced germinal center kinase-related (GCKR) and stress-activated protein kinase (SAPK) activation depends upon the E2/E3 complex Ubc13-Uev1A/TNF receptor-associated factor 2 (TRAF2). J. Biol. Chem..

[219] Ea C.K., Deng L., Xia Z.P., Pineda G., Chen Z.J. (2006). Activation of IKK by TNFalpha requires site-specific ubiquitination of RIP1 and polyubiquitin binding by NEMO. Mol. Cell.

[220] Wu K., Kovacev J., Pan Z.Q. (2010). Priming and extending: A UbcH5/Cdc34 E2 handoff mechanism for polyubiquitination on a SCF substrate. Mol. Cell.

[221] Liu S., Chen Z.J. (2011). Expanding role of ubiquitination in NF-κB signaling. Cell Res..

[222] Zhang Y., Li Y., Yang X., Wang J., Wang R., Qian X., Zhang W., Xiao W. (2018). Uev1A-Ubc13 catalyzes K63-linked ubiquitination of RHBDF2 to promote TACE maturation. Cell. Signal..

[223] Shao L., Liu Y., Wang W., Li A., Wan P., Liu W., Shereen M.A., Liu F., Zhang W., Tan Q. (2020). SUMO1 SUMOylates and SENP3 deSUMOylates NLRP3 to orchestrate the inflammasome activation. FASEB J..

[224] Hoesel B., Schmid J.A. (2013). The complexity of NF-κB signaling in inflammation and cancer. Mol. Cancer.

[225] Dolcet X., Llobet D., Pallares J., Matias-Guiu X. (2005). NF-kB in development and progression of human cancer. Virchows Arch..

[226] Prasad S., Ravindran J., Aggarwal B.B. (2010). NF-kappaB and cancer: How intimate is this relationship. Mol. Cell Biochem..

[227] Xiao W., Lin S.L., Broomfield S., Chow B.L., Wei Y.F. (1998). The products of the yeast MMS2 and two human homologs (hMMS2 and CROC-1) define a structurally and functionally conserved Ubc-like protein family. Nucleic Acids Res..

[228] Syed N.A., Andersen P.L., Warrington R.C., Xiao W. (2006). Uev1A, a ubiquitin conjugating enzyme variant, inhibits stress-induced apoptosis through NF-kappaB activation. Apoptosis.

[229] Wu Z., Shen S., Zhang Z., Zhang W., Xiao W. (2014). Ubiquitin-conjugating enzyme complex Uev1A-Ubc13 promotes breast cancer metastasis through nuclear factor-κB mediated matrix metalloproteinase-1 gene regulation. Breast Cancer Res..

[230] Dynek J.N., Goncharov T., Dueber E.C., Fedorova A.V., Izrael-Tomasevic A., Phu L., Helgason E., Fairbrother W.J., Deshayes K., Kirkpatrick D.S. (2010). c-IAP1 and UbcH5 promote K11-linked polyubiquitination of RIP1 in TNF signalling. EMBO J..

[231] Ditsworth D., Zong W.X. (2004). NF-kappaB: Key mediator of inflammation-associated cancer. Cancer Biol. Ther..

[232] Xiong Y., Yi Y., Wang Y., Yang N., Rudd C.E., Liu H. (2019). Ubc9 Interacts with and SUMOylates the TCR Adaptor SLP-76 for *NFAT* Transcription in T Cells. J. Immunol..

[233] Hattori K., Hatakeyama S., Shirane M., Matsumoto M., Nakayama K. (1999). Molecular dissection of the interactions among IkappaBalpha, FWD1, and Skp1 required for ubiquitin-mediated proteolysis of IkappaBalpha. J. Biol. Chem..

[234] Yamoah K., Oashi T., Sarikas A., Gazdoiu S., Osman R., Pan Z.Q. (2008). Autoinhibitory regulation of SCF-mediated ubiquitination by human cullin 1’s C-terminal tail. Proc. Natl. Acad. Sci. USA.

[235] Vallabhapurapu S., Matsuzawa A., Zhang W., Tseng P.H., Keats J.J., Wang H., Vignali D.A., Bergsagel P.L., Karin M. (2008). Nonredundant and complementary functions of TRAF2 and TRAF3 in a ubiquitination cascade that activates NIK-dependent alternative NF-kappaB signaling. Nat. Immunol..

[236] Xiao G., Harhaj E.W., Sun S.C. (2001). NF-kappaB-inducing kinase regulates the processing of NF-kappaB2 p-100. Mol. Cell.

[237] Ghosh S., May M.J., Kopp E.B. (1998). NF-kappa B and Rel proteins: Evolutionarily conserved mediators of immune responses. Annu. Rev. Immunol..

[238] Senftleben U., Cao Y., Xiao G., Greten F.R., Krähn G., Bonizzi G., Chen Y., Hu Y., Fong A., Sun S.C. (2001). Activation by IKKalpha of a second, evolutionary conserved, NF-kappa B signaling pathway. Science.

[239] Wu Z., Niu T., Xiao W. (2019). Uev1A promotes breast cancer cell survival and chemoresistance through the AKT-FOXO1-BIM pathway. Cancer Cell Int..

[240] Hao P., Kang B., Li Y., Hao W., Ma F. (2019). UBE2T promotes proliferation and regulates PI3K/Akt signaling in renal cell carcinoma. Mol. Med. Rep..

[241] Duncan L.M., Piper S., Dodd R.B., Saville M.K., Sanderson C.M., Luzio J.P., Lehner P.J. (2006). Lysine-63-linked ubiquitination is required for endolysosomal degradation of class I molecules. EMBO J..

[242] Shukla S., Allam U.S., Ahsan A., Chen G., Krishnamurthy P.M., Marsh K., Rumschlag M., Shankar S., Whitehead C., Schipper M. (2014). KRAS protein stability is regulated through SMURF2: UBCH5 complex-mediated β-TrCP1 degradation. Neoplasia.

[243] Vila I.K., Yao Y., Kim G., Xia W., Kim H., Kim S.J., Park M.K., Hwang J.P., González-Billalabeitia E., Hung M.C. (2017). A UBE2O-AMPKα2 Axis that Promotes Tumor Initiation and Progression Offers Opportunities for Therapy. Cancer Cell.

[244] Wang L., Ji S. (2019). Inhibition of Ubc9-Induced CRMP2 SUMOylation Disrupts Glioblastoma Cell Proliferation. J. Mol. Neurosci..

[245] Vij R., Wang M., Kaufman J.L., Lonial S., Jakubowiak A.J., Stewart A.K., Kukreti V., Jagannath S., McDonagh K.T., Alsina M. (2012). An open-label, single-arm, phase 2 (PX-171-004) study of single-agent carfilzomib in bortezomib-naive patients with relapsed and/or refractory multiple myeloma. Blood.

[246] Pal A., Young M.A., Donato N.J. (2014). Emerging potential of therapeutic targeting of ubiquitin-specific proteases in the treatment of cancer. Cancer Res..

[247] Hewitt W.M., Lountos G.T., Zlotkowski K., Dahlhauser S.D., Saunders L.B., Needle D., Tropea J.E., Zhan C., Wei G., Ma B. (2016). Insights into the Allosteric Inhibition of the SUMO E2 Enzyme Ubc9. Angew. Chem. Int. Ed. Engl..

[248] Ramatenki V., Dumpati R., Vadija R., Vellanki S., Potlapally S.R., Rondla R., Vuruputuri U. (2017). Targeting the ubiquitin-conjugating enzyme E2D4 for cancer drug discovery-a structure-based approach. J. Chem. Biol..

[249] Wang L., Zhang L. (2020). MicroRNAs in amyotrophic lateral sclerosis: From pathogenetic involvement to diagnostic biomarker and therapeutic agent development. Neurol. Sci..

[250] Chen Y., Gao D.Y., Huang L. (2015). In vivo delivery of miRNAs for cancer therapy: Challenges and strategies. Adv. Drug Deliv. Rev..

[251] Sharma P., Dando I., Strippoli R., Kumar S., Somoza A., Cordani M., Tafani M. (2020). Nanomaterials for Autophagy-Related miRNA-34a Delivery in Cancer Treatment. Front. Pharmacol..

[252] Liu L., Hua Y., Wang D., Shan L., Zhang Y., Zhu J., Jin H., Li H., Hu Z., Zhang W. (2014). A sesquiterpene lactone from a medicinal herb inhibits proinflammatory activity of TNF-α by inhibiting ubiquitin-conjugating enzyme UbcH5. Chem. Biol..

[253] Chen H., Wu G., Gao S., Guo R., Zhao Z., Yuan H., Liu S., Wu J., Lu X., Yuan X. (2017). Discovery of Potent Small-Molecule Inhibitors of Ubiquitin-Conjugating Enzyme UbcH5c from α-Santonin Derivatives. J. Med. Chem..

[254] Xu Y., Meng X. (2020). Molecular Simulation Elaborating the Mechanism of 1β-Hydroxy Alantolactone Inhibiting Ubiquitin-Conjugating Enzyme UbcH5s. Sci. Rep..

[255] Wang C., Shi G., Ji X. (2018). Design, synthesis, and anticancer activity evaluation of irreversible allosteric inhibitors of the ubiquitin-conjugating enzyme Ube2g2. Med. Chem. Commun..

[256] Sanders M.A., Brahemi G., Nangia-Makker P., Balan V., Morelli M., Kothayer H., Westwell A.D., Shekhar M.P.V. (2013). Novel inhibitors of Rad6 ubiquitin conjugating enzyme: Design, synthesis, identification, and functional characterization. Mol. Cancer Ther..

[257] Ceccarelli D.F., Tang X., Pelletier B., Orlicky S., Xie W., Plantevin V., Neculai D., Chou Y.C., Ogunjimi A., Al-Hakim A. (2011). An allosteric inhibitor of the human Cdc34 ubiquitin-conjugating enzyme. Cell.

[258] Kim Y.S., Keyser S.G., Schneekloth J.S. (2014). Synthesis of 2′,3′,4′-trihydroxyflavone (2-D08), an inhibitor of protein sumoylation. Bioorg. Med. Chem. Lett..

[259] Zlotkowski K., Hewitt W.M., Sinniah R.S., Tropea J.E., Needle D., Lountos G.T., Barchi J.J., Waugh D.S., Schneekloth J.S. (2017). A Small-Molecule Microarray Approach for the Identification of E2 Enzyme Inhibitors in Ubiquitin-Like Conjugation Pathways. SLAS Discov..

[260] Tsukamoto S., Takeuchi T., Rotinsulu H., Mangindaan R.E., van Soest R.W., Ukai K., Kobayashi H., Namikoshi M., Ohta T., Yokosawa H. (2008). Leucettamol A: A new inhibitor of Ubc13-Uev1A interaction isolated from a marine sponge, Leucetta aff. microrhaphis. Bioorg. Med. Chem. Lett..

[261] Ushiyama S., Umaoka H., Kato H., Suwa Y., Morioka H., Rotinsulu H., Losung F., Mangindaan R.E., de Voogd N.J., Yokosawa H. (2012). Manadosterols A and B, sulfonated sterol dimers inhibiting the Ubc13-Uev1A interaction, isolated from the marine sponge Lissodendryx fibrosa. J. Nat. Prod..

[262] Cheng J., Fan Y.H., Xu X., Zhang H., Dou J., Tang Y., Zhong X., Rojas Y., Yu Y., Zhao Y. (2014). A small-molecule inhibitor of UBE2N induces neuroblastoma cell death via activation of p53 and JNK pathways. Cell Death Dis..

[263] Lin T.H., Hsu W.H., Tsai P.H., Huang Y.T., Lin C.W., Chen K.C., Tsai I.H., Kandaswami C.C., Huang C.J., Chang G.D. (2017). Dietary flavonoids, luteolin and quercetin, inhibit invasion of cervical cancer by reduction of UBE2S through epithelial-mesenchymal transition signaling. Food Funct..

[264] Cornwell M.J., Thomson G.J., Coates J., Belotserkovskaya R., Waddell I.D., Jackson S.P., Galanty Y. (2019). Small-Molecule Inhibition of UBE2T/FANCL-Mediated Ubiquitylation in the Fanconi Anemia Pathway. ACS Chem. Biol..

[265] Jin D., Guo J., Wu Y., Du J., Wang X., An J., Hu B., Kong L., Di W., Wang W. (2020). Retraction of “*UBE2C*, Directly Targeted by miR-548e-5p, Increases the Cellular Growth and Invasive Abilities of Cancer Cells Interacting with the EMT Marker Protein Zinc Finger E-box Binding Homeobox 1/2 in NSCLC”. Theranostics.

[266] Lu J., Gu X., Liu F., Rui Z., Liu M., Zhao L. (2019). Antitumor effects of hsa-miR661-3p on non-small cell lung cancer in vivo and in vitro. Oncol. Rep..

[267] Hu J., Wu X., Yang C., Rashid K., Ma C., Hu M., Ding Q., Jiang H. (2019). Anticancer effect of icaritin on prostate cancer via regulating miR-381-3p and its target gene *UBE2C*. Cancer Med..

[268] Wei X., You X., Zhang J., Zhou C. (2019). MicroRNA-1305 Inhibits the Stemness of LCSCs and Tumorigenesis by Repressing the UBE2T-Dependent Akt-Signaling Pathway. Mol. Ther. Nucleic Acids.

[269] Zhao Z., Tan X., Zhao A., Zhu L., Yin B., Yuan J., Qiang B., Peng X. (2012). microRNA-214-mediated *UBC9* expression in glioma. BMB Rep..

